# Silencing *Srsf6* does not modulate incomplete splicing of the huntingtin gene in Huntington’s disease models

**DOI:** 10.1038/s41598-020-71111-w

**Published:** 2020-08-20

**Authors:** Michael A. Mason, Casandra Gomez-Paredes, Kirupa Sathasivam, Andreas Neueder, Aikaterini-Smaragdi Papadopoulou, Gillian P. Bates

**Affiliations:** 1 Huntington’s Disease Centre, Department of Neurodegenerative Disease and UK Dementia Research Institute at UCL, Queen Square Institute of Neurology, University College London, London, WC1N 3BG UK; 2 Department of Neurology, Ulm University, 89081 Ulm, Germany

**Keywords:** Molecular biology, Neuroscience, Diseases, Neurology

## Abstract

We have previously shown that the incomplete splicing of exon 1 to exon 2 of the *HTT* gene results in the production of a small polyadenylated transcript (*Httexon1*) that encodes the highly pathogenic exon 1 HTT protein. There is evidence to suggest that the splicing factor SRSF6 is involved in the mechanism that underlies this aberrant splicing event. Therefore, we set out to test this hypothesis, by manipulating SRSF6 levels in Huntington’s disease models in which an expanded CAG repeat had been knocked in to the endogenous *Htt* gene. We began by generating mice that were knocked out for *Srsf6*, and demonstrated that reduction of SRSF6 to 50% of wild type levels had no effect on incomplete splicing in zQ175 knockin mice. We found that nullizygosity for *Srsf6* was embryonic lethal, and therefore, to decrease SRSF6 levels further, we established mouse embryonic fibroblasts (MEFs) from wild type, zQ175, and zQ175::*Srsf6*^+/−^ mice and transfected them with an *Srsf6* siRNA. The incomplete splicing of *Htt* was recapitulated in the MEFs and we demonstrated that ablation of SRSF6 did not modulate the levels of the *Httexon1* transcript. We conclude that SRSF6 is not required for the incomplete splicing of *HTT* in Huntington’s disease.

## Introduction

Huntington’s disease is a monogenic neurodegenerative disorder that manifests with psychiatric, motor and cognitive symptoms^1^. It is caused by a CAG repeat expansion mutation in the first exon of the huntingtin (*HTT*) gene^2^, which encodes an expanded polyglutamine tract in the HTT protein. There are currently no effective treatments to delay the onset or slow the progression of the disease and current efforts are largely focused on knocking down *HTT* mRNA^3–6^. This is a particularly attractive target as it is upstream of the pathogenic HTT protein, and therefore, understanding how the *HTT* mRNA is processed in the context of Huntington’s disease could inform future therapeutic strategies.

Both human *HTT* and mouse *Htt* are known to be transcribed into five mature protein-coding mRNAs: three endogenous 67 exon full-length transcripts that differ by the length of their 3′ UTRs and encode a 350 kDa protein^7,8^ and, in the context of an expanded CAG repeat, two transcripts that contain exon 1 and intron 1 sequences (*HTTexon1*), and are translated to produce the exon 1 HTT protein^9^. The *HTTexon1* mRNA is generated by the incomplete splicing of exon 1 to exon 2 of the *HTT* gene and the aberrant activation of one of two cryptic polyadenylation (polyA) signals in intron 1 resulting in premature termination. These cryptic polyA signals are located at 680 and 1,145 bp into intron 1 for mouse *Htt*^9,10^ and 2,710 and 7,327 bp into intron 1 for human *HTT*^11^. We have previously shown that *HTTexon1* is present in Huntington’s disease patient *post*-*mortem* brains and fibroblast lines^11^ as well as all Huntington’s disease mouse models that contain a mutant version of the complete mouse or human gene^12,13^. The exon 1 HTT protein, that this small transcript encodes, is highly toxic^14,15^ and aggregation-prone^16^.

The RNA processing mechanisms that result in the production of *Httexon1* have yet to be elucidated^17^. Expanded CAG repeats within RNA form hairpin loops^18^ and are known to sequester a number of RNA binding proteins in a CAG repeat-dependent fashion^18,19^. In other microsatellite repeat disorders such as amyotrophic lateral sclerosis—frontal temporal dementia^20^ and myotonic dystrophy^21^, functional sequestration of RNA binding proteins by expanded polynucleotide repeat RNA is known to impact cellular RNA processing events and lead to pathogenesis. It is therefore plausible that the incomplete splicing of *HTT* is caused by sequestration of RNA-binding proteins in a CAG repeat-dependent fashion. Bioinformatic analysis indicated that the predicted recognition sequence for serine/arginine-rich splicing factor 6 (SRSF6) included a CAG repeat, and an SRSF6 antibody immunoprecipitated greater levels of 5′ *Htt* RNA sequences from the zQ175 knockin brain lysates than from wild type mice^9^. That SRSF6 has a greater binding affinity to *HTTexon1* with an expanded CAG repeat than to *HTTexon1* with a CAG repeat in the normal range was supported by an independent study^19^. Finally, we showed that incomplete splicing of a mouse *Htt* minigene decreased following SRSF6 knockdown by RNA interference and increased following SRSF6 overexpression in cell culture^10^. SRSF6 is a member of the serine/arginine-rich (SR) family of proteins, an evolutionary conserved group defined by one or two N-terminal RNA-binding domains and a C-terminal SR domain, which mediates protein–protein interactions^22,23^. SR proteins mainly localise to the nucleus but some also shuttle between the nucleus and cytoplasm^24^. They all function as constitutive and alternative splicing factors but have also been shown to operate in a number of co-transcriptional and co-translational processes.

In this study, we set out to investigate whether SRSF6 reduction modulated *Htt* splicing in vivo. To do this, a constitutive *Srsf6* knockout mouse model was generated and nullizygosity for *Srsf6* was found to be embryonic lethal. Therefore, heterozygous *Srsf6*^+/−^ mice were bred to the zQ175 knockin mouse model of Huntington’s disease to examine the effect of decreasing SRSF6 to 50% of wild type levels. This was found to have no effect on the levels of *Httexon1* in various zQ175 brain regions. We then derived mouse embryonic fibroblasts (MEFs) from the progeny of the *Srsf6*^+/−^ x zQ175 cross to investigate the effects of further lowering SRSF6 levels by RNA interference. We found that the incomplete splicing of *Htt* was recapitulated in the MEFs and that SRSF6 ablation had no effect on the incomplete splicing of *Htt.* We conclude that SRSF6 levels do not play a role in the mechanism that underlies this aberrant splicing event.

## Results

### Characterisation of the *Srsf6* knockout mouse model

**Figure 1:**
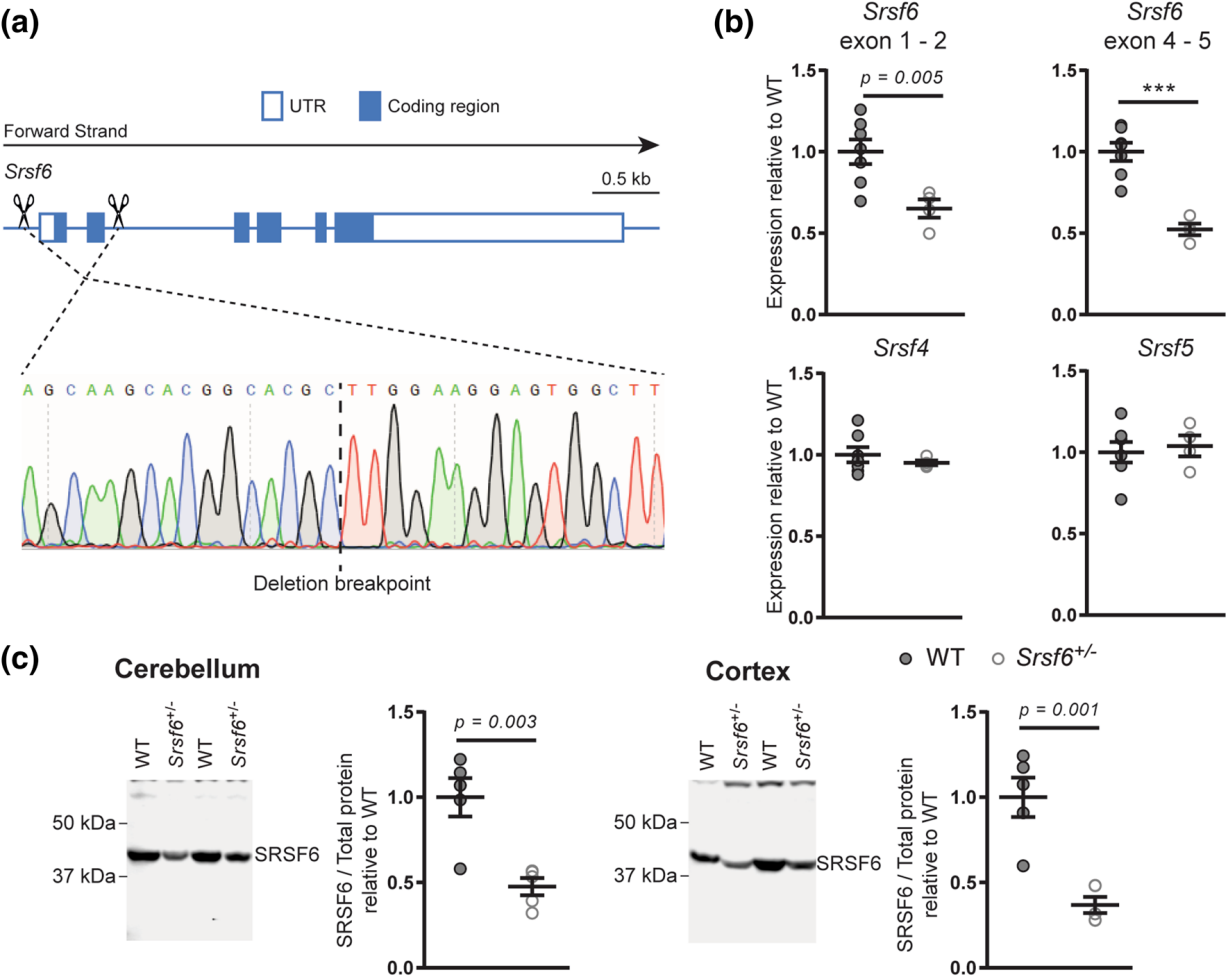
Characterisation of the *Srsf6* knockout mouse line. (**a**) Schematic of mouse *Srsf6* with scissors denoting approximate Cas9 cleavage sites used to generate the knockout allele. Sanger sequencing was used to confirm the deletion breakpoint for the Δ990 bp line. *UTR* untranslated region. (**b**) qPCR analysis showed *Srsf6* mRNA levels to be 50% of WT whereas, *Srsf4* or *Srsf5* levels were unchanged in cortex from 2 month old *Srsf6*^+*/−*^ Δ990 mice. n = 7 WT and 4 *Srsf6*^+*/−*^ mice. (**c**) Western blot analysis showed a 50% reduction in SRSF6 in cerebellum and cortex from 2 month old *Srsf6*^+*/−*^ mice compared to WT littermates. See Supplementary Fig. S7 for uncropped blots and total protein loading controls. n = 5/genotype. Statistical analyses were by unpaired Student’s *t*-tests. Test statistics can be found in Supplementary Table S4. *WT* wild type.

In order to study the effect of SRSF6 reduction on the incomplete splicing of *Htt*, *Srsf6* knockout mice were generated by the Jackson Laboratory using CRISPR/Cas9. Guide RNAs targeting sequences upstream of the 5′ untranslated region and within intron 2 were used to generate the *Srsf6* knockout alleles (Fig. 1a), giving rise to four founder lines. We used TaqMan real-time quantitative PCR (qPCR) assays to measure cortical transcripts encoding SR proteins in two of the founder lines with deletion sizes of 990 bp and 956 bp. mRNA levels were measured for *Srsf6* and also *Srsf4* and *Srsf5*, the two paralogs with the greatest RNA and protein sequence homology^22^. To determine whether transcriptional inactivation of the *Srsf6* knockout allele had occurred, we used two *Srsf6* assays: one at the boundary of exon 1–2 (corresponding to part of the deleted sequence of the knockout allele) and the other at the boundary of exon 4–5 (which remained intact on the knockout allele). The transcripts for the SR proteins were normalised to *Canx*, *Ubc* and *Atp5b* as reference genes. We found that *Srsf6* levels were 50% of wild type (WT) in both heterozygous *Srsf6* strains (*Srsf6*^+/−(Δ990bp)^ and *Srsf6*^+/−(Δ956bp)^) using both the exon 1–2 and exon 4–5 assays (Fig. 1b, Supplementary Fig. S1). This implies that transcription of the knockout allele had been inactivated. There were no significant differences in *Srsf4* and *Srsf5* levels in either of the heterozygous *Srsf6* founder strains compared to WT indicating that the *Srsf6* paralogs are not upregulated to compensate for the deleted gene (Fig. 1b, Supplementary Fig. S1). Since we saw no differences between the two founder lines at the mRNA level, we performed all succeeding experiments with the *Srsf6*^+/−*(Δ990bp)*^ mice, which shall be denoted *Srsf6*^+*/*−^ from this point forward. The deletion breakpoint was confirmed by DNA sequencing (Fig. 1a) and we verified that the promoter of *Srsf6*, as predicted by the eukaryotic promoter database^25^, had been removed. We observed a concurrent two-fold reduction in SRSF6 protein levels in cortex and cerebellum of *Srsf6*^+*/*−^ compared to WT (Fig. 1c).

To assess the viability of homozygous *Srsf6* knockout mice (*Srsf6*^−*/*−^), we intercrossed *Srsf6*^+*/*−^ heterozygous mice. From the twenty-eight progeny, we observed that nine (32.1%) were WT, nineteen (67.9%) were *Srsf6*^+*/*−^ and none were *Srsf6*^−*/*−^ homozygotes (Table 1). We confirmed that our observed distribution was significantly different from the expected distribution assuming that *Srsf6*^−*/*−^ mice were viable (*χ*^2^(2) = 9.357, *p* = 0.009) but not significantly different from the expected distribution assuming that *Srsf6*^−*/*−^ mice were inviable (*χ*^2^(1) = 0.018, *p* = 0.895) (Table 1). Therefore, we conclude that homozygosity for *Srsf6* knockout is embryonically lethal in mice and all in vivo experiments going forward utilised *Srsf6*^+*/*−^ heterozygous mice.

**Table 1:** Genotypes of the progeny from the *Srsf6*^+/*−*^ intercross.

Genotype	Progeny observed	Percentage	Hypothesis 1 expected values (*Srsf6*^*−/−*^* viable*)	Hypothesis 2 expected values (*Srsf6*^*−/−*^* inviable*)
WT	9 (4 m, 5f.)	32.1%	7 (25%)	9.33 (33.3%)
Srsf6^+/−^	19 (9 m, 10f.)	67.9%	14 (50%)	18.66 (66.7%)
Srsf6^−/−^	0	0%	7 (25%)	0 (0%)
Statistics				
Χ^2^			9.357	0.018
Degrees of freedom			2	1
p-value			0.009	0.895
Statistical significance?			Yes—distributions different	No—distributions the same

### Heterozygosity for *Srsf6* knockout does not change *Htt* splicing patterns in zQ175 mouse brain

**Figure 2:**
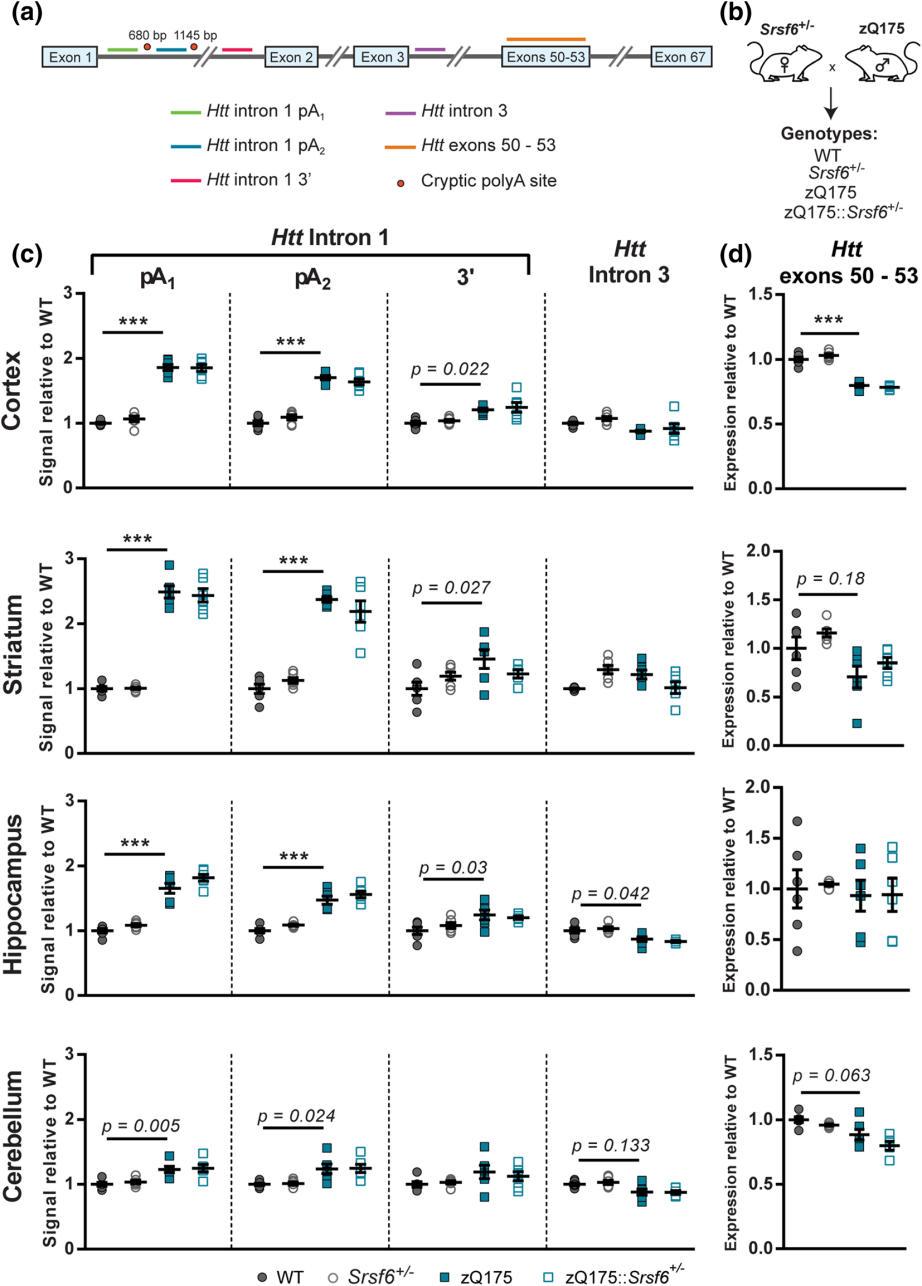
QuantiGene analysis of *Htt* transcripts in brain regions from the progeny of the zQ175 and *Srsf6*^+*/−*^ mouse cross. (**a**) Schematic of the location of the QuantiGene plex probe sets on the mouse *Htt* transcript. (**b**) *Srsf6*^+*/−*^ female mice were bred to zQ175 knockin male mice to generate progeny with four genotypes: WT, *Srsf6* heterozygous knockout (*Srsf6*^+*/−*^), zQ175 knockin and double mutants (zQ175::*Srsf6*^+*/−*^). (**c**) *Httexon1* was detected in the cortex, striatum, hippocampus and cerebellum of 2 month old zQ175 mice but was not altered by heterozygosity for *Srsf6* knockout. (**d**) Full-length *Htt* was measured in the cortex, striatum, hippocampus and cerebellum using the *Htt* exon 50–53 assay. Cortical full-length *Htt* was significantly lower in zQ175 mice compared to WT and this was not changed by heterozygosity for *Srsf6* knockout. n = 6/genotype. Statistical analysis was by one-way ANOVA with Bonferroni correction for multiple pairwise comparisons, ***p < 0.001, *p* < 0.2 values are indicated. Test statistics can be found in Supplementary Table S5. *WT* wild type.

We used the zQ175 knockin model of Huntington’s disease to investigate the effect of heterozygous *Srsf6* knockout on incomplete splicing. The zQ175 mice were generated by replacing mouse *Htt* exon 1 with human *HTT* exon 1 with an expanded CAG repeat^26–28^, and the *Httexon1* transcript can be readily detected in this model using a previously designed high-throughput QuantiGene plex assay^29^. The QuantiGene plex included the following probe sets: five *Htt*, *Srsf4*, *Srsf5*, two *Srsf6* and a number of housekeeping reference genes (Supplementary Table S1). Four of the five *Htt* targets were intronic sequences and the fifth spanned exons 50–53, which measured the level of the processed full-length *Htt* transcript (Fig. 2a). Three intronic *Htt* targets were in intron 1: before the first cryptic polyA signal (*Htt* intron 1 pA_1_), between the two cryptic polyA signals (*Htt* intron 1 pA_2_) and at the 3′ end of intron 1 (*Htt* intron 1 3′) (Fig. 2a). *Htt* intron 1 pA_1_ detected the *Httexon1* mRNA which terminated at the first or second polyA signal and *Htt* intron 1 *Htt* pA_2_ targets *Httexon1* terminated at the second polyA signal. *Htt* intron 1 3′ and *Htt* intron 3 detected unprocessed mRNAs that contained intron 1 or intron 3 sequences (Fig. 2a).

To investigate the effect of SRSF6 reduction on incomplete splicing, we bred *Srsf6*^+/−^ mice to zQ175 knockins, generating four genotypes: WT, zQ175, *Srsf6*^+/−^ and zQ175::*Srsf6*^+/−^ (Fig. 2b). The QuantiGene plex was used to measure the levels of the SR protein transcripts and determine the effect of SRSF6 reduction on *Htt* and *Httexon1* transcripts in cortex, striatum, hippocampus and cerebellum from 2 month-old mice. As expected, *Srsf6* was reduced to 50% of WT levels in both the *Srsf6*^+/−^ and zQ175::*Srsf6*^+/−^ mice whereas *Srsf4* and *Srsf5* were unchanged (Supplementary Fig. S2). The *Httexon1* transcript was present in all brain regions in zQ175 mice, with the highest levels detected in striatum and the lowest levels detected in cerebellum (Fig. 2c), consistent with previous data^29^. The level of the *Httexon1* transcript detected by the *Htt* intron 1 pA_2_ probe set was comparable to that detected by *Htt* intron 1 pA_1_, suggesting that the majority of *Httexon1* terminated at the second cryptic polyA site (Fig. 2c). The reduction of SRSF6 in zQ175 mice had no effect on the level of *Httexon1*. Comparison of the levels of the 3′ end of intron 1 and intron 3 in the four genotypes, revealed small changes, with an increase in intron 1 and a decrease in intron 3 in some brain regions, possibly reflecting effects of the mutation on *Htt* mRNA processing (Fig. 2c).

The level of the full-length *Htt* transcript was decreased in cortex, which may reflect a combination of incomplete splicing and a reduction in transcription as a consequence of the expanded CAG repeat^30^ (Fig. 2d). In contrast to our previous data, we did not detect a reduction in full-length *Htt* in the striatum and hippocampus^29^. The exon 50–53 probe set produced more variable data than probe sets for full-length *Htt* that we have designed to the 3′UTR^29^, which we would recommend using for future studies. Heterozygosity for *Srsf6* in WT mice did not affect the levels of full-length *Htt* in any of these brain regions (Fig. 2d).

### *Httexon1* can be detected in zQ175 mouse embryonic fibroblasts (MEFs)

**Figure 3:**
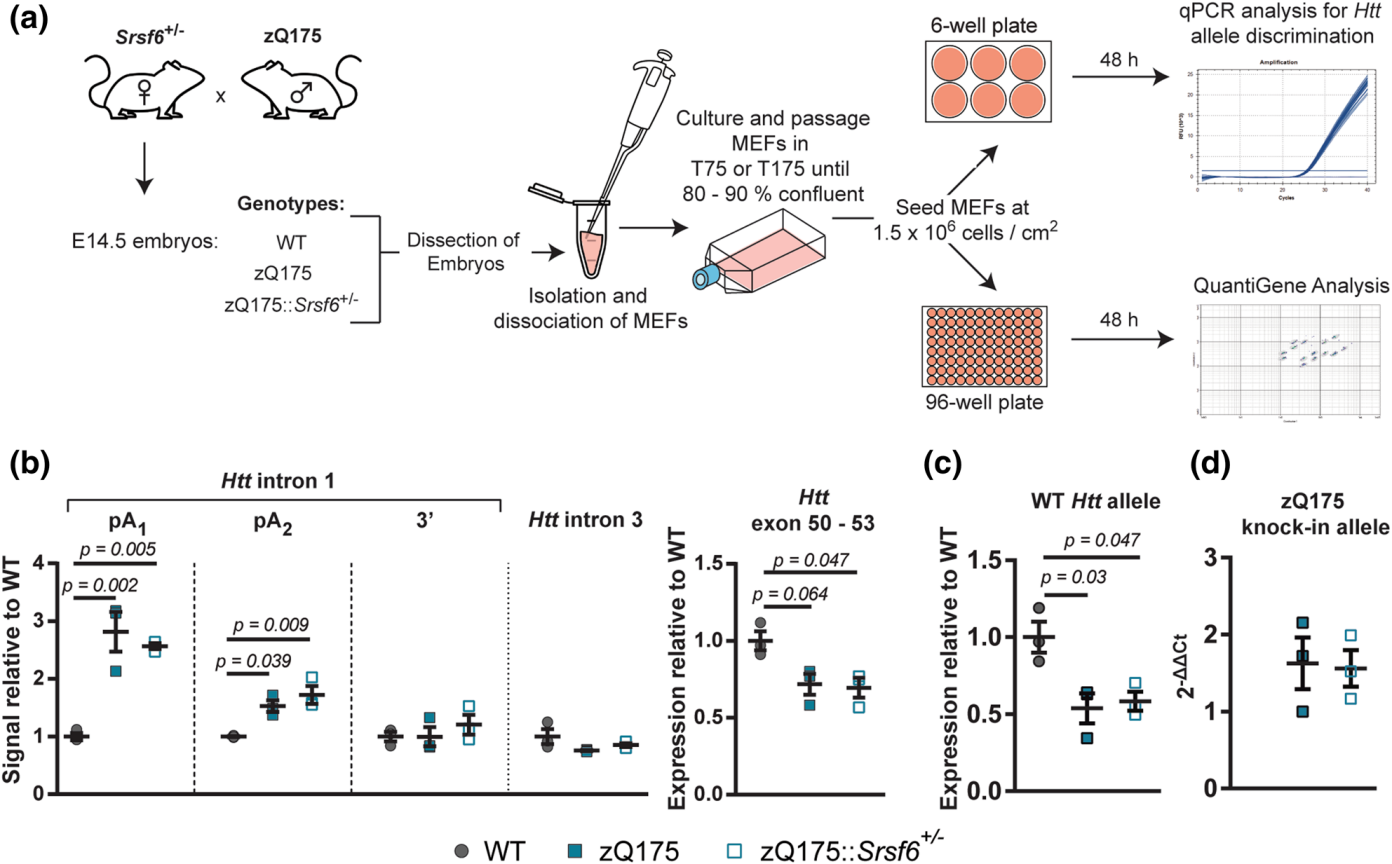
Generation and characterisation of zQ175 mouse embryonic fibroblasts (MEFs). (**a**) Schematic shows workflow for derivation and characterisation of MEF cell cultures. *Srsf6*^+*/−*^ mice were bred to zQ175 mice. The female was sacrificed at approximately E14.5 and embryos were dissected. Mouse embryonic fibroblasts (MEFs) were isolated, cultured, passaged and expanded as required. MEFs were seeded for qPCR or QuantiGene assays as required. (**b**) QuantiGene analysis showed that *Httexon1* was present in both the zQ175 and zQ175::*Srsf6*^+*/−*^ MEFs at comparable levels, and that full-length *Htt* was decreased in both the zQ175 and zQ175::*Srsf6*^+/−^ cells to a similar degree. n = 3 biological replicates/genotype. Statistical analysis was by one-way ANOVA with Bonferroni correction for multiple pairwise comparisons, ***p < 0.001. Test statistics can be found in Supplementary Table S6. (**c**,**d**) *Htt* allele discrimination qPCRs were used to measure the (**c**) WT and (**d**) zQ175 knockin *Htt* alleles in the MEF lines. n = 3 biological replicates/genotype. Statistical analysis was by unpaired Student’s *t*-test or one-way ANOVA with Bonferroni correction for multiple pairwise comparisons, ***p < 0.001, *p* < 0.2 values are indicated. Test statistics can be found in Supplementary Table S7.

We sought to generate a cell model in which *Httexon1* mRNA could be detected. zQ175 males were bred to *Srsf6*^+/−^ females, E14.5 embryos were harvested and mouse embryonic fibroblasts (MEFs) from these embryos were isolated and cultured (Fig. 3a). QuantiGene analysis demonstrated that *Httexon1* mRNA could be detected in the zQ175 and zQ175::*Srsf6*^+/−^ MEFs at comparable levels (Fig. 3b), consistent with our in vivo data. Most of the *Httexon1* transcript terminated at the first cryptic polyA signal, with much lower levels extending to the second cryptic polyA site (Fig. 3b). Full-length *Htt* levels were reduced by approximately 30% in both zQ175 and zQ175::*Srsf6*^+/−^ MEFs compared to WT (Fig. 3b). To investigate whether this reduction in *Htt* had occurred for both the WT and knockin alleles, we employed a qPCR assay that specifically detected WT full-length *Htt*. We found that the level of WT *Htt* in the zQ175 and zQ175::*Srsf6*^+/−^ MEFs, was 50% of that in the WT MEFs (Fig. 3c). Therefore, the observed decrease in full-length *Htt* in the zQ175 and zQ175::*Srsf6*^+/−^ MEFs represented a reduction in the levels of the knockin allele by approximately 60% of WT *Htt* levels. We next used a qPCR assay that specifically targeted the full-length knockin *Htt* allele, and demonstrated that this is present at equivalent levels in the zQ175 and zQ175::*Srsf6*^+/−^ MEFs (Fig. 3d). For the SR protein genes: *Srsf6* levels were 50% lower in zQ175::*Srsf6*^+/−^ compared to WT or zQ175 MEFs, *Srsf4* was increased by about 1.2-fold, contrary to the situation in all brain regions except striatum (Supplementary Fig. S2), and there was no change in *Srsf5* levels (Supplementary Fig. S3). Taken together, we show that zQ175 MEFs express detectable and quantifiable levels of *Httexon1* and that, as we observed in brain, *Httexon1* and full-length *Htt* mRNA levels are unaffected by heterozygosity for *Srsf6* knockout.

### Silencing SRSF6 does not alter incomplete *Htt* splicing in zQ175 MEFs

**Figure 4:**
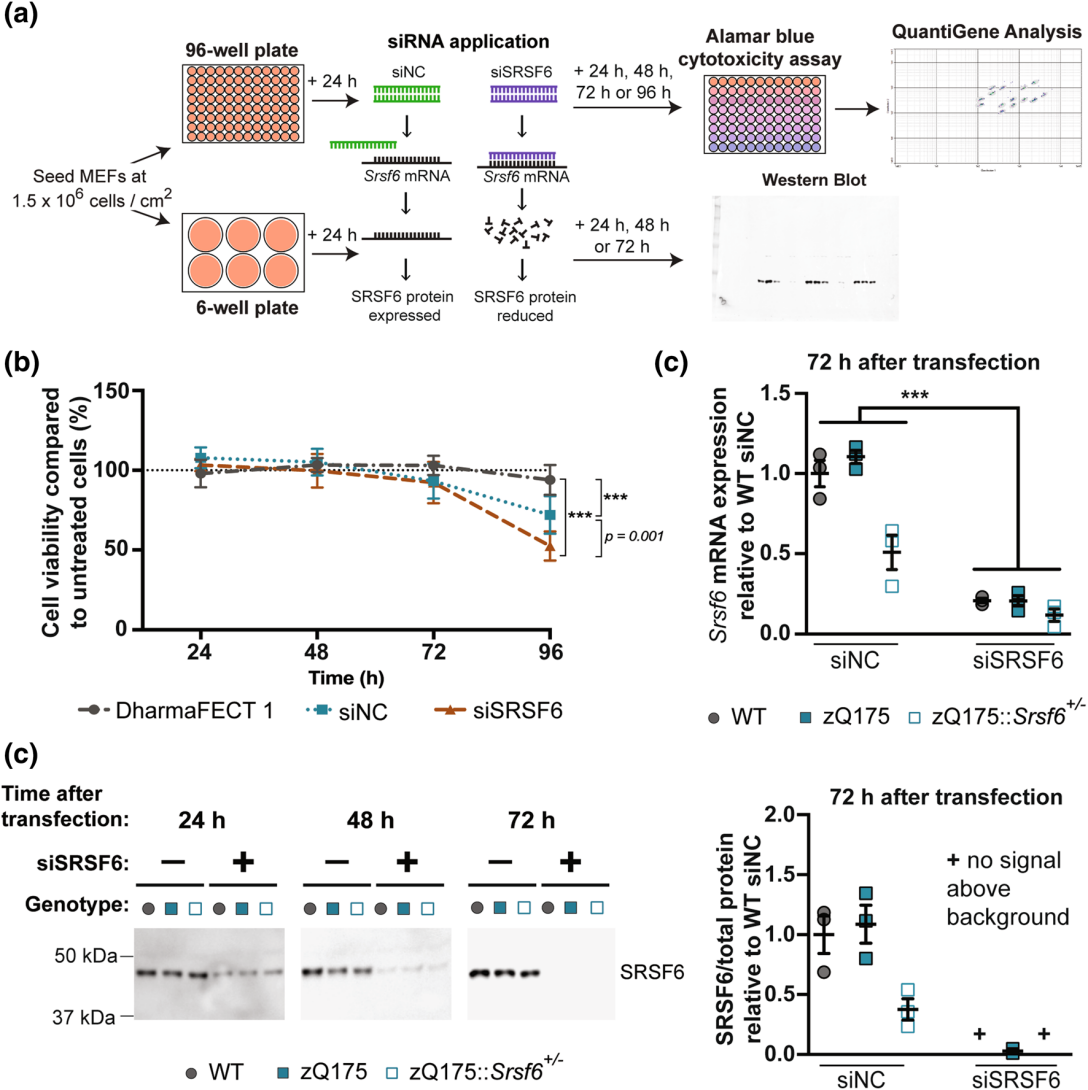
RNA interference experimental plan, cytotoxicity of MEFs, *Srsf6* transcript and SRSF6 protein quantification. (**a**) Schematic showing experimental workflow for RNA interference experiments in WT, zQ175 and zQ175::*Srsf*6^+/*−*^ MEFs. MEFs were seeded and transfected with either an *Srsf6-*targeting (siSRSF6) siRNA or a non-targeting negative control (siNC) using DharmaFECT 1 transfection reagents and harvested after 24, 48, 72 or 96 h. Cells were used in alamarBlue, QuantiGene or western blot experiments. (**b**) Cytotoxicity was measured using the alamarBlue assay. The viability was calculated as the signal from siSRSF6 or siNC transfected cells as a percentage of the signal from DharmaFect 1 vehicle-treated MEFs. n = 9 cell cultures/treatment group. Data were analysed by one-way ANOVAs, ***p < 0.001. Test statistics can be found in Supplementary Table S8. Data stratified by genotype can be found in Supplementary Fig. S4. (**c**) QuantiGene analysis showed that *Srsf6* mRNA levels decreased to 80–90% of that in the siNC-treated cells by 72 h post transfection. n = 3 biological replicates/genotype. Statistical analysis was by two-way ANOVA, ***p < 0.001. Test statistics can be found in Supplementary Table S9. (**d**) Western blots of the SRSF6 protein in MEFs 72 h after siSRSF6 or siNC transfection. To quantify SRSF6 levels, the SRSF6 signal was normalised to a total protein loading control. SRSF6 protein levels are plotted relative to WT. n = 3 biological replicates/genotype. Uncropped blots and total protein loading controls in Supplementary Figs. S8, S9 and S10.

As knocking out a single allele of *Srsf6* did not affect *Htt* mRNA processing in zQ175 mouse brain or MEFs, we used RNA interference to investigate whether further SRSF6 reduction in the zQ175 MEFs might alter *Httexon1* mRNA levels (Fig. 4a). An siRNA targeting *Srsf6* (siSRSF6) and a non-targeting negative control (siNC) were selected, transfected into WT, zQ175 and zQ175::*Srsf6*^+/−^ MEFs using DharmaFECT 1, and cell viability was assessed using the alamarBlue assay after 24, 48, 72 and 96 h post-transfection. Neither siSRSF6 nor siNC were cytotoxic as compared to vehicle control (DharmaFect 1 transfection reagent) up to 72 h but both were cytotoxic to the MEFs by 96 h (Fig. 4b, Supplementary Fig. S4). *Srsf6* levels were reduced to around 80–90% 72 h after transfection with siSRSF6 as compared to siNC (Fig. 4c, Supplementary Fig. S5). Western blotting showed that although *Srsf6* mRNA levels were not completely silenced, SRSF6 protein was ablated by 72 h (Fig. 4d). This is likely because siRNAs degrade mRNA via cytoplasmic P-bodies^31^ and therefore nuclear *Srsf6* mRNA may be preserved. This experiment confirmed that *Srsf4* mRNA was increased in MEFs heterozygous for *Srsf6* knockout at 24 h post-transfection (Supplementary Fig. S5), but this was not apparent at later time points. At 48 h post-transfection, there was a modest increase in *Srsf5* levels (1.1-fold) in MEFs heterozygous for *Srsf6* knockout, and reduction of the *Srsf6* transcripts by 80–90% resulted in a modest increase in *Srsf5* mRNA in wild type and zQ175 MEFs (Supplementary Fig. S5).

**Figure 5:**
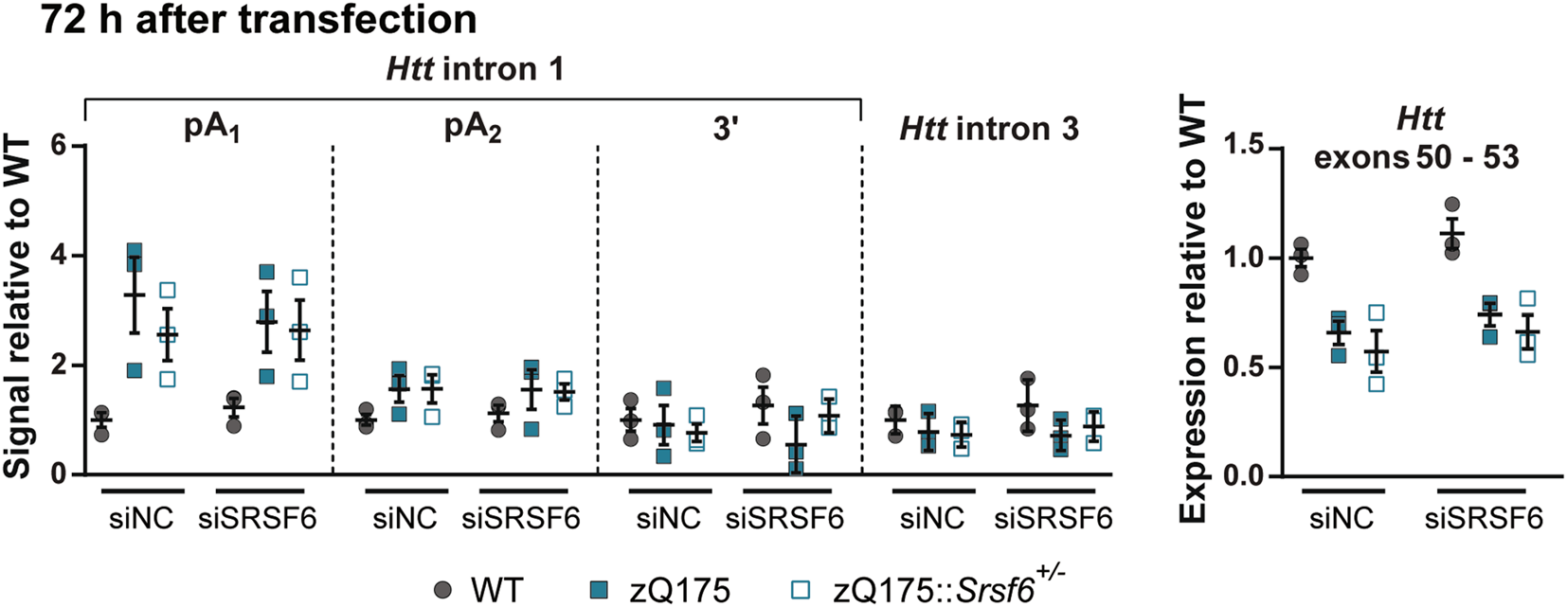
Measurement of *Httexon1* and *Htt* transcripts in WT, zQ175 and zQ175::*Srsf6*^+/*−*^ MEFs after transfection with an siRNA targeting *Srsf6* (siSRSF6). QuantiGene analysis was used to measure *Httexon1* and *Htt* mRNA levels 72 h after siSRSF6 or siNC transfection. Neither *Httexon1* nor full-length *Htt* levels were changed in MEFs treated with siSRSF6 compared to siNC. n = 3 biological replicates/genotype. Statistical analysis was by two-way ANOVA, ***p < 0.001. Test statistics can be found in Supplementary Table S10.

Despite the dramatic reduction in SRSF6 protein levels, culminating in its ablation after 72 h post-transfection with siSRSF6, we saw no change in the levels of *Httexon1* or full-length *Htt* in the zQ175 or zQ175::*Srsf6*^+/−^ MEFs as compared to those that had been transfected with siNC (Fig. 5, Supplementary Fig S6).

## Discussion

Our previous data suggested that the splicing factor SRSF6 might play a critical role in the mechanism that, in the context of expanded CAG repeats, underlies the incomplete splicing of the *HTT* gene resulting in the production of the highly pathogenic exon 1 HTT protein^9,10^. In order to validate this hypothesis in vivo, we generated *Srsf6* knockout mice and demonstrated that a 50% reduction in the levels of SRSF6 did not alter the production of the *Httexon1* transcript in brain regions from the zQ175 knockin mouse model of Huntington’s disease. We found that MEFs derived from the zQ175 mice provided a very useful cell culture system in which methods to modulate the splicing of exon 1 to exon 2 of *Htt* can be tested. Using an siRNA to *Srsf6*, to ablate SRSF6 in the MEFs, we were able to show that the absence of SRSF6 has no effect on the incomplete splicing of *Htt*.

We generated constitutive *Srsf6* knockout mice to investigate the role of SRSF6 in the incomplete splicing of *Htt* in the context of the endogenous gene in vivo. We found that heterozygosity for *Srsf6* resulted in a 50% reduction in *Srsf6* mRNA and SRSF6 protein levels throughout the brain. *Srsf4* and *Srsf5* are the two paralogs with the greatest gene and protein sequence alignment to *Srsf6*^22^. *Srsf4* and *Srsf5* brain levels were comparable between the wild type and *Srsf6*^+/−^ knock-out mice, indicating that there was no biological compensatory upregulation. This is also consistent with the report showing that the removal of the promoter in the knockout design ensures that compensatory expression of paralogous genes or homologous alleles, due to genetic manipulation, does not occur at the RNA level^32^. We were unable to generate any viable homozygous *Srsf6* knockout mice, implying that SRSF6 plays a critical role in mammalian embryonic development. Similarly, genetic ablation of *Srsf1*, *Srsf2* and *Srsf3* are all embryonic lethal in mice^33–35^.

We previously designed a QuantiGene assay to measure the level of *Httexon1* and full-length *Htt* transcripts in brain regions from zQ175 knockin mice^29^, and used this to compare the levels of these *Htt* transcripts in the brains of wild type, zQ175 and zQ175::*Srsf6*^+/−^ mice. Consistent with our previous data, the *Httexon1* transcript predominantly terminated at the second cryptic polyA site and the highest levels were detected in striatum, followed by cortex, hippocampus and the lowest levels were in cerebellum. We observed 20% lower level of full-length *Htt* in zQ175 cortex compared to wild type, which could be a consequence of incomplete splicing and a decrease in transcription through the expanded CAG repeat. The QuantiGene probe set exons 50–53 used here for full-length *Htt* gave quite variable data for the other three brain regions, and we would instead recommend using probe sets to the 3′UTR that we published in Papadopoulou et al.^29^ for this purpose. From this analysis, we were able to conclude that heterozygosity for *Srsf6* knockout had no effect on *Httexon1* or full-length *Htt* levels in the zQ175 brain.

We established MEFs from wild type, zQ175 and zQ175::*Srsf6*^+/−^ E14.5 embryos to see whether these might provide a cell culture system that could be used to explore the effects of further reducing *Srsf6* levels on the levels of the *Httexon1* and full-length *Htt* transcripts in the context of the knockin allele. The QuantiGene assay was optimised for cells grown in 96-well plates and we found that the *Httexon1* transcript could be readily detected. In contrast to the zQ175 brain regions, *Httexon1* mostly terminated at the first cryptic polyA site. We saw a concurrent reduction in full-length *Htt* to approximately 70% of wild type, and using allele specific qPCR assays, showed that this corresponds to a reduction in the mutant and not wild type transcript. As in brain, we found that heterozygosity for *Srsf6* knockout did not alter the levels of *Httexon1* or full-length *Htt*. Having validated the MEFs as a useful model, we used an siRNA targeting the *Srsf6* transcript (siSRSF6) to further reduce SRSF6 in zQ175 MEFs. We found that the *Srsf6* transcript was reduced by up to 90% and the SRSF6 protein was completely ablated 72 h after transfection. Unlike the situation in zQ175 brain, heterozygosity for *Srsf6* knockout resulted in modest increases in *Srsf4* at 24 h, and *Srsf5* at 48 h post-transfection. Ablation of SRSF6 had no effect on the incomplete splicing of *Htt* in the zQ175 MEFs.

Our interest in SRSF6 originated from the bioinformatic prediction that it binds to CAG and CAGCAA repeats, and therefore we proposed a model in which its ectopic location might sequester the U1 spliceosome complex, locally decreasing the probability of exon 1 splicing to exon 2 at the same time exposing cryptic polyA sites within intron 1^10^. In this study, we have conclusively shown that SRSF6 is not required for the incomplete splicing of *Htt* in models in which the CAG expansion occurs in the context of the endogenous gene. Although, in the MEFs, we saw a slight increase in the levels of *Srsf4* and *Srsf5*, these splicing factors are not predicted to bind CAGs, and therefore, any compensatory roles that they are playing are likely occurring at the transcriptome-level, and not in a context relevant to *Htt* splicing. The factors that bind to *Htt* mRNA and influence either the efficiency of splicing and/or the probability of premature termination are currently unknown. A number of RNA-binding proteins have been shown to bind *Htt* mRNA^19^ and the zQ175 MEFs will provide a powerful resource for screening candidates that may influence incomplete splicing of *Htt*, thereby increasing our understanding of this mechanism. The MEFs will also be invaluable for screening therapeutics that specifically target the *HTTexon1* transcript that encodes the highly pathogenic exon 1 HTT protein.

## Methods

### Generation of the *Srsf6* knockout mouse lines

*Srsf6* knockout mice were generated by the Jackson Laboratory (Bar Harbor, Maine, USA) using CRIPSR/Cas9. Guide RNAs targeted the 5′UTR (AGCAAGCACGGCACGCGCCG and GGGCCGCTCCGGATGTGTTG) and intron 2 (CCCCGCGCTCCGGTGTCCCA and CCGTGGGACACCGGAGCGCG). Four founders with different deletions were generated on a C57BL/6J background and preliminary experiments were conducted on two of the four strains (Δ990 bp and Δ956 bp).

### Animal colony maintenance and breeding

All animal procedures were undertaken in compliance with the Animals (Scientific Procedures) Act 1986 with the authorisation of the University College London Ethical Review Process Committee. zQ175 and *Srsf6*^+*/*−^ colonies were maintained by backcrossing males to C57BL6/J females (Charles River). To generate the zQ175 x *Srsf6*^+*/*−^ cross, zQ175 males were mated with *Srsf6*^+*/*−^ females. Mice had unrestricted access to food and water, were provided with environmental enrichment which included chew sticks and play tubes, and were maintained under a 12 h light/dark cycle. The animal facility was barrier-maintained and quarterly non-sacrificial FELASA screens found no evidence of pathogens. Two month-old mice were sacrificed, dissected, and their tissues were snap frozen in liquid nitrogen and stored at − 80 °C.

### DNA extractions, genotyping and CAG repeat sizing

To isolate genomic DNA, ear notches and tails from 10 day-old mice and E14.5 embryos respectively were lysed overnight in 500 μL of lysis solution (0.05 M TRIS–HCl pH 8.0, 0.1 M EDTA, 0.5% SDS and 0.5 mg/mL proteinase K). 300 μL of saturated 36% (w/v) NaCl was thoroughly mixed with lysates and centrifuged at 1.7 × 10^5^ g for 30 min. The supernatant was thoroughly mixed with 650 μL of 100% ethanol in a fresh Eppendorf tube and centrifuged at 1.7 × 10^5^ g for 20 min. The supernatant was removed and the pellet was washed by addition of 200 μL 70% ethanol followed by centrifugation at 1.7 × 10^5^ *g* for 5 min. The supernatant was removed and pellets were air dried at RT for 2 h. Pellets were re-suspended in 100 μL of 5 mM TRIS–HCl pH 8. Genomic DNA was quantified using a Nanodrop (Thermo Fisher Scientific).

Mice from the zQ175 colony were genotyped using a forward primer (AGGAGCCGCTGCACCGA) and a reverse primer (CTCTTCACAACAGTCATGTGCG). The PCR thermal cycling program was as follows: 98 °C for 30 s followed by 35 cycles of 98 °C for 15 s, 64 °C for 15 s and 72 °C for 30 s, and lastly 72 °C for 5 min. Mice from the *Srsf6* knockout colony were genotyped using a forward primer (GCGTGTACTCAACGAAACCA) and two reverse primers (GCTGTCAGTCTAGGCCATCT for the WT allele and AGCCTCCCAGCTCCTAAGAC for the knockout allele). The PCR thermal cycling program was as follows: 94 °C for 3 min followed by 35 cycles of 96 °C for 30 s, 64 °C for 30 s and 72 °C for 30 s and lastly 72 °C for 5 min. Genotyping PCRs were performed using the GoTaq system (Promega). PCR amplicons and 100 bp DNA size standard ladder (New England BioLabs) were electrophoresed in 2% agarose gels containing 0.003% SYBR Safe (Thermo Fisher Scientific) and imaged using a Gel Doc XR (BioRad). The zQ175 PCR yielded a WT band of 324 bp and a knockin band of 240 bp. The *Srsf6* PCR yielded a WT band of 104 bp and a knockout band of 516 bp.

CAG repeat sizing was performed using a 6-FAM-labelled forward primer (ATGAAGGCCTTCGAGTCCCTCAAGTCCTTC) and a reverse primer (GGCGGCTGAGGAAGCTGAGGA). The PCR thermal cycling program was as follows: 94 °C for 90 s, then 35 cycles of 94 °C for 30 s, 65 °C for 30 s and 72 °C for 90 s, and lastly 72 °C for 10 min. The CAG repeat sizing PCR was performed using the AmpliTaq system (Thermo Fisher Scientific). 2 μL of DNA amplicon was mixed with 8 μL of HiDi Formamide (Thermo Fisher Scientific) and 0.05 μL of MapMarker Rox 1000 (Bioventures) in a 96-well plate. DNA was denatured at 95 °C for 10 min then rapidly cooled on ice for 10 min protected from light. Data were acquired by capillary electrophoresis on a 3,730 DNA analyser (Applied Biosystems) and repeat sizing was analysed using GeneMarker software (SoftGenetics). The CAG repeat size for the zQ175 mice was 203.39 ± 4.97 (SD), for zQ175::*Srsf6*^+*/*−^ mice was 204.28 ± 4.89 (SD), for the zQ175 MEFs was 202 ± 3.61 (SD) and for the zQ175::*Srsf6*^+/−^ MEFs was 205.33 ± 3.12 (SD).

### DNA sequencing for *Srsf6* knockout deletion breakpoint

The *Srsf6* knockout allele was amplified by the genotypic PCR protocol and the band was excised. The amplicon was extracted using a Qiagen gel extraction kit (Qiagen) as per the manufacturer’s instructions. DNA was quantified using a Qubit fluorometer (Thermo Fisher Scientific). To generate fragments for sequencing, 200 ng of the extracted amplicon was added to a BigDye Terminator V Mastermix (Thermo Fisher Scientific) as follows: 0.5 μL of Big Dye v3.1 terminator (Thermo Fisher Scientific), 2 μL of 5 × Big Dye Sequencing Buffer (Thermo Fisher Scientific), 3.2 μM of a primer in a final volume of 10 μL. Forward primers were GCTGAAGGGAAAGAGCAACC, CCTGGGCACAAGAACAGTTT and GCGAAATCAACTCCCAGCAA and reverse primers were ACCCCAGCCTTCCTAGAAAC, CCTCGCTTTCAATGGCAGAA and CCCAAGTCAGTGCCAAAGAG. PCR reactions were prepared in a 96-well plate and were run using the following thermal cycling program: 96 °C for 2 min, then 30 cycles of 96 °C for 30 s, 50 °C for 15 s and 60 °C for 2 min. Following the thermal cycling, 30 μL of 100% ethanol and 2.5 μL of 125 mM EDTA pH 8.0 was added to each sample mixed by vortexing and incubated at RT protected from light. The plate was centrifuged at 1 × 10^4^ *g* for 40 min. The supernatant was removed by inverting the plate and replaced with 50 μL of 70% ethanol. The plate was vortexed and centrifuged at 1 × 10^4^ *g* for 30 min and the supernatant removed. The plate was then centrifuged upside down on a paper towel at 6.5 × 10^3^ *g* for 1 min. The pellets were re-suspended in 10 µL of HiDi formamide and denatured at 95 °C for 10 min then rapidly cooled on ice for 10 min protected from light. Data were acquired by capillary electrophoresis by a 3,730 DNA analyser (Applied Biosystems) and analysed using SnapGene software (SoftGenetics).

### Cell culture, RNA interference (RNAi) and alamarBlue cytotoxicity assay

Mouse embryonic fibroblasts (MEFs) were isolated from E14.5 embryos from a cross between zQ175 and *Srsf6*^+*/*−^ mice. MEFs were maintained in DMEM/10% FBS/1% penicillin–streptomycin at 37 °C in 5% CO_2_ and passaged roughly once a week when MEFs were around 90% confluent.

MEFs were seeded in 6-well plates (for western blot or qPCR analysis) or 96-well plates (for AlamarBlue or QuantiGene analysis) at a density of 1.5 × 10^6^ cells/cm^2^ in DMEM/6% FBS. RNAi experiments were performed one day post-seeding when the medium was changed to DMEM/OptiMEM (in a 1:1 ratio)/3% FBS before siRNA transfection. 10 nM of Silencer Select siRNAs (Thermo Fisher Scientific) targeting *Srsf6* (assay ID: s86053) and a non-targeting negative control siRNA (assay ID: 4390843) were transfected into MEFs using 2.5 nL of DhramaFECT 1 transfection reagent (Horizon Discovery) per μL of total media in each well as per the manufacturer’s instructions.

Cytotoxicity of siRNAs was assessed using the AlamarBlue cytotoxicity assay (Thermo Fisher Scientific). The media was aspirated and replaced with 90 μL of fresh DMEM/3% FBS and 10 μL of the AlamarBlue reagent was added to each well followed by an incubation at 37 °C in 5% CO_2_ for 3 h. Absorbance was measured using a SPECTROstar Nano Microplate Reader (BMG Labtech). Media containing AlamarBlue reagent was aspirated, replaced with 100 μL of DMEM/3% FBS and prepared for QuantiGene analysis (see following section).

For MEFs in 6-well plates, the media was aspirated and the cells were washed once with PBS. For western blot analysis: 250 μL Trypsin–EDTA 0.25% (Thermo Fisher scientific) was applied and incubated at 37 °C in 5% CO_2_ for 3 min to dislodge the MEFs from the bottom of the well. 500 μL of DMEM/3% FBS was added to each well to neutralise the trypsin and the cell suspension was added to a fresh 1.5 mL centrifuge tube. MEFs were pelleted by centrifugation at 500*g* at RT. The supernatant aspirated and the pellet washed in ice-cold PBS by centrifugation at 500*g* at 4 °C. The supernatant was aspirated and the pellets were snap-frozen on dry ice and processed for western blot analysis (see following section). For qPCR experiments: Qiazol (Qiagen) was added to each well, the MEFs were scraped from the bottom of the well and the lysate was transferred to a fresh 1.5 mL centrifuge tube. Cells were processed for qPCR (see section below).

### RNA isolation and cDNA synthesis

Cortex was homogenised in Qiazol (Qiagen) using a 1 mL syringe with a 23G followed by an 18G needle. Total RNA was isolated from cortex and MEFs using an RNeasy mini-kit (Qiagen) according to the manufacturer’s instructions. This included a DNase step using an RNase-Free DNase Set (Qiagen) as per the manufacturer’s instructions. RNA was eluted in 30 μL of nuclease-free water (Sigma) and quantified using a Nanodrop (Thermo Fisher Scientific). cDNA was synthesised by reverse transcription from 1 μg of RNA using M-MLV Reverse Transcriptase (Invitrogen) and an oligo-dT_(18)_ primer (Invitrogen) as per the manufacturer’s instructions. A reverse transcription negative control was included.

### Real-time quantitative PCR (qPCR) assays

To quantify specific transcripts, commercially available Taqman real-time quantitative PCR (qPCR) assays (Thermo Fisher Scientific) were used to measure genes of interest and housekeeping genes (Supplementary Table S2). We also used proprietary Taqman qPCR assays targeting the WT^29^ and zQ175 knockin *Htt* alleles separately. For the knockin allele, the forward primer was GCCCGGCTGTGGCTGA, the reverse primer was TTCACACGGTCTTTCTTGGTGG and the ZEN probe was TGCACCGACCAAAGAAGGAACTCT (Integrated DNA Technologies). cDNA was diluted 1:50 nuclease-free water (Sigma) and plated in 96-well thin wall Hard-Shell PCR plates (BioRad). Each 15 μL reaction per sample contained 1 × Taqman Fast Advanced Mastermix (Thermo Fisher Scientific), 1 × Taqman Gene expression assays and 3 μL of diluted cDNA (1:50 in nuclease-free water) and was aliquoted into the 96-well plates and sealed. Plates were centrifuged at 8 × 10^3^ *g* for 30 s and then analysed using a BioRad CFX96 thermal cycler with the following program: 95 °C for 40 s, followed by 40 cycles of 95 °C for 7 s, 60 °C for 20 s. All biological replicates were run in triplicate. C_q_ values deviating by ± 0.25 from the mean of the triplicate were removed from the analysis. Data for genes of interest were normalised to reference genes (*CanX*, *Ubc* and *Atp5b*) as per the 2^-ΔΔCt2^ method^36^.

### Tissue lysis and QuantiGene assays

Brain samples were prepared by lysis in 60 μL of QuantiGene homogenising solution (Thermo Fisher Scientific) and 30 μg proteinase K (Thermo Fisher Scientific) per mg of tissue using a 1 mL syringe with a 23G followed by a 18G needle in a 1.5 mL microfuge tube. Lysates were incubated at 50 °C on a dry heat block for 18 h and then centrifuged at 1.7 × 10^5^ *g* for 10 min. The supernatant was transferred to a fresh 1.5 mL microfuge tube, snap frozen on dry ice and stored at − 80 °C until required. Lysates were thawed on a heat block at 50 °C for 30 min and diluted (for tissue specific dilutions, refer to Supplementary Table S3). Cell lysates were prepared by adding 50 μL of QuantiGene Lysis Mixture containing 25 μg of proteinase K to each 96-well of a cell culture plate (which contained 100 μL of cell culture medium per well) and pipetting up and down three to four times. 96-well plates containing lysates were snap frozen on dry ice and stored at − 80 °C until required. Prior to analysis, QuantiGene cell lysates were incubated at 50 °C for 1 h and lysis was confirmed under a light microscope. Cell lysates were transferred to 1.5 mL centrifuge tubes and centrifuged at RT at 1.7 × 10^5^ *g* for 10 min to pellet any debris. The QuantiGene plex assay was run in duplicate as per the manufacturer’s instructions except that the Streptavidin *R*-Phycoerythrin conjugate (SAPE) was incubated at 51 °C. After subtraction of the background, the median fluorescence intensity (MFI) for the genes of interest was normalised to the geometric mean of the MFI for the reference genes (See Supplementary Table S1 for QuantiGene probe sets).

### Western Blot

Tissue or cell lysates were prepared in ice-cold KCL buffer (50 mM Tris–HCl pH8, 10% Glycerol, 5 mM EDTA, 150 mM KCl, 1 mM DTT, 1 mM PMSF) containing cOmplete proteinase inhibitor cocktail (Sigma-Aldrich) and kept on ice throughout the entire lysis process. Tissues were sheared in KCl buffer using a 1 mL syringe with a 23G and then an 18G needle. Cell pellets were re-suspended and samples were sonicated using a Q125 Sonicator (Qsonica) six times for 10 s with a 10 s break between each sonication at an amplitude setting of 40%. Debris was removed by centrifugation at 4 °C at 1.7 × 10^5^ *g* and lysates were stored at − 80 °C until required. Protein was quantified using Pierce BCA Protein Assay (Thermo Fisher Scientific). 40 μg or 15 μg of protein in Laemmli loading buffer from brain tissue or cells respectively was denatured at 90 °C for 5 min and resolved on Criterion TGX Stain-Free precast gels (BioRad) by electrophoresis at 13.5 V/cm^2^. Total protein was measured directly from the Criterion TGX Stain-Free precast gels by UV transillumination of the blot for 2.5 min using a Gel Doc XR + transilluminator (BioRad). Protein was transferred to a 0.45 μm nitrocellulose membrane (Biorad) by submerged transfer in transfer buffer (25 mM Tris base, 192 mM glycine, 20% v/v methanol). The membrane was blocked in PBS/0.1% TWEEN 20 (PBST)/5% milk at RT for 1 h and then incubated with anti-SRSF6 (Ab140623; Abcam) at a dilution of 1:1,000 at 4 °C overnight in PBS/0.1% TWEEN 20 (PBST)/1% milk. Blots were washed four times for 5 min in PBS/0.1% TWEEN 20 (PBST) and then incubated with 1:5,000 HRP-conjugated goat anti-rabbit (A31460; Invitrogen) at RT for 1 h. Blots were washed four times for 5 min in PBS/0.1% TWEEN 20. Clarity Western ECL Substrate (BioRad) was applied to the membranes and images were acquired using a ChemiDoc (BioRad). Blots were analysed using Image Lab software (BioRad).

### Statistical analysis

Data were screened for outliers using ROUT test (GraphPad Software, California, USA) and any outliers were removed before between-group comparisons. Statistical analysis was performed with SPSS v26 (IBM, Portsmouth, UK) using two-tailed independent samples Student’s *t*-test, one-way ANOVA or two-way ANOVA with Bonferroni *post-hoc* tests as indicated. Graphs were prepared using Prism v7 (GraphPad Software, California, USA). A significance threshold of α = 0.05 was set for all statistical tests.

## Supplementary information

Supplementary Information.

## Data Availability

The datasets generated and/or analysed during the current study are available from the corresponding author on reasonable request.
