# Supplementary material for: Silencing Srsf6 does not modulate incomplete splicing of the huntingtin gene in Huntington’s disease models
Source: Sci Rep. 2020 Aug 20;10:14057. doi: 10.1038/s41598-020-71111-w (PMC7441155; doi:10.1038/s41598-020-71111-w)
Supplement: Supplementary file 1 — Supplementary Information. [file 41598_2020_71111_MOESM1_ESM.pdf]

Silencing SRSF6 does not modulate incomplete splicing of the huntingtin gene in Huntington's disease models

Michael A. Mason[1], Casandra Gomez-Paredes[1], Kirupa Sathasivam[1], Andreas Neueder[2], Aikaterini-Smaragdi Papadopoulou[1] and Gillian P. Bates[1]*

[1]Huntington's Disease Centre, Department of Neurodegenerative Disease and UK Dementia Research Institute at UCL, Queen Square Institute of Neurology, University College London, London WC1N 3BG, UK.

[2]Department of Neurology, Ulm University, 89081 Ulm, Germany

*Corresponding Author: gillian.bates@ucl.ac.uk

Supplementary Information

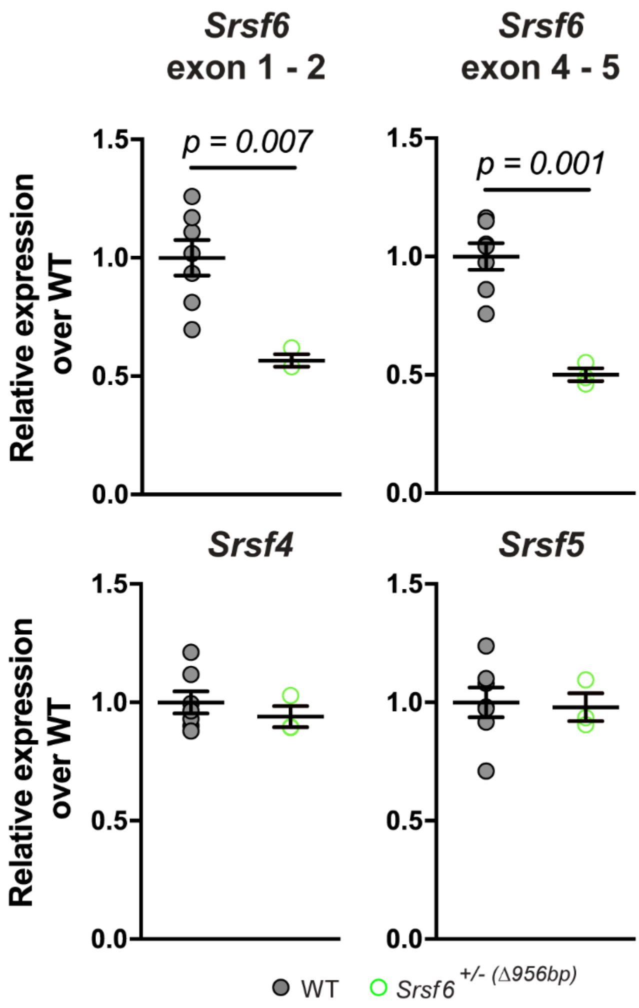

Supplementary Figure S1. SR transcript levels in the *Srsf6* heterozygous knockout founder strain Δ956. qPCR was used to measure cortical levels of *Srsf6* in the *Srsf6*$^{+/-}$ founder with the 956 bp deletion. *Srsf6* levels were approximately 50% lower than WT and *Srsf4* and *Srsf5* levels remained unchanged. Statistical analyses were by unpaired Student's *t*-test, $^{***}p < 0.001$. Test statistics can be found in Supplementary Table S11.

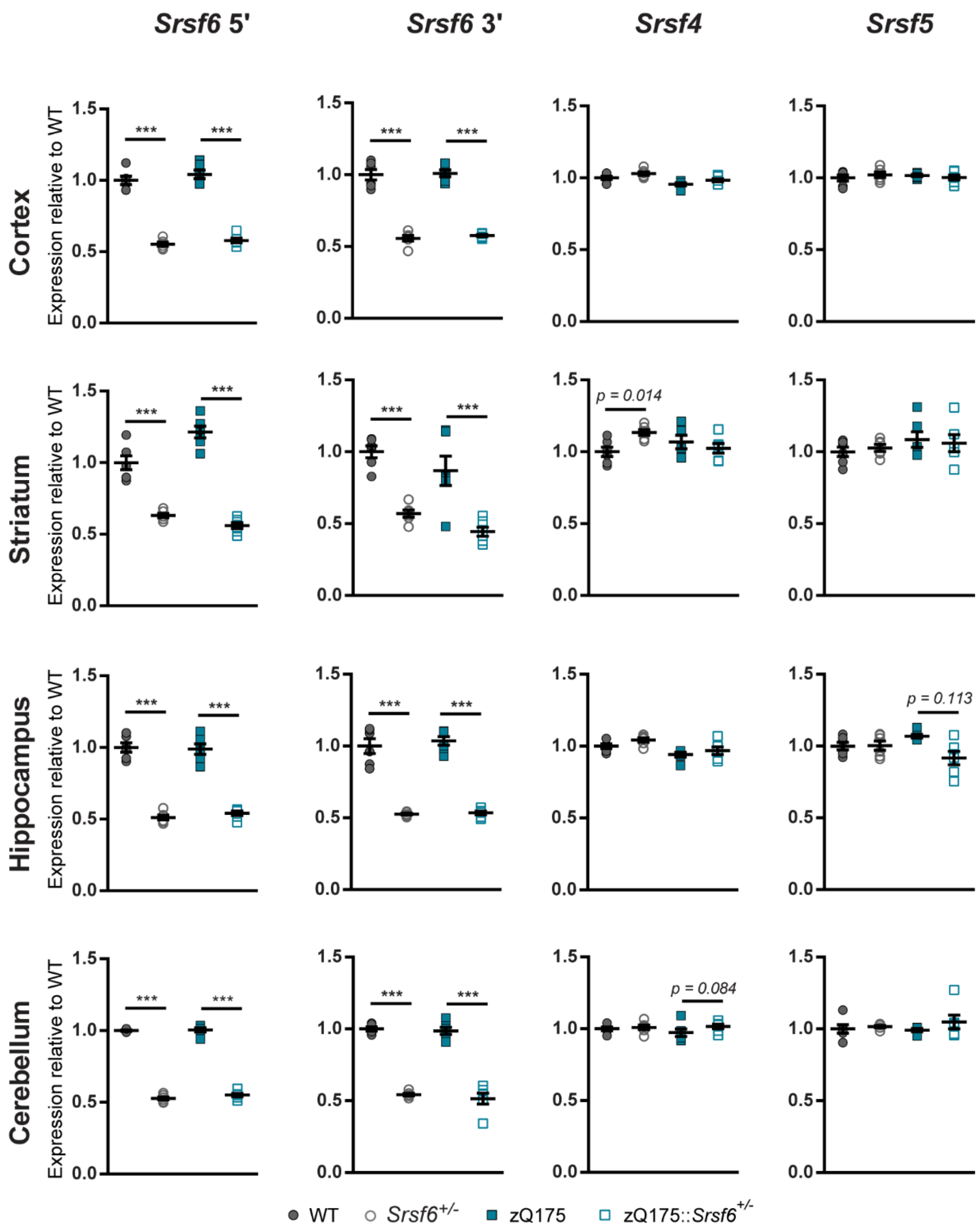

Supplementary Figure S2. Transcripts for SR proteins were measured in 2 month old mouse brain regions by QuantiGene. *Srsf6* levels were measured using probes targeting the 5' end of the gene and the 3' end of the gene in cortex, striatum, hippocampus and cerebellum. *Srsf6* levels were 50 % lower in *Srsf6*$^{+/-}$ and zQ175::*Srsf6*$^{+/-}$ mice compared to WT and zQ175 respectively. *Srsf4* and *Srsf5* transcript levels were not significantly different between any of the genotypes apart from *Srsf4* in striatum, where a 1.2-fold increase in *Srsf6*$^{+/-}$ mice compared to WT was observed. n = 6/genotype. Data were analysed by one-way ANOVA with Bonferroni correction for multiple pairwise comparisons, ***p<0.001, p < 0.2 values are indicated. Test statistics can be found in Supplementary Table S12.

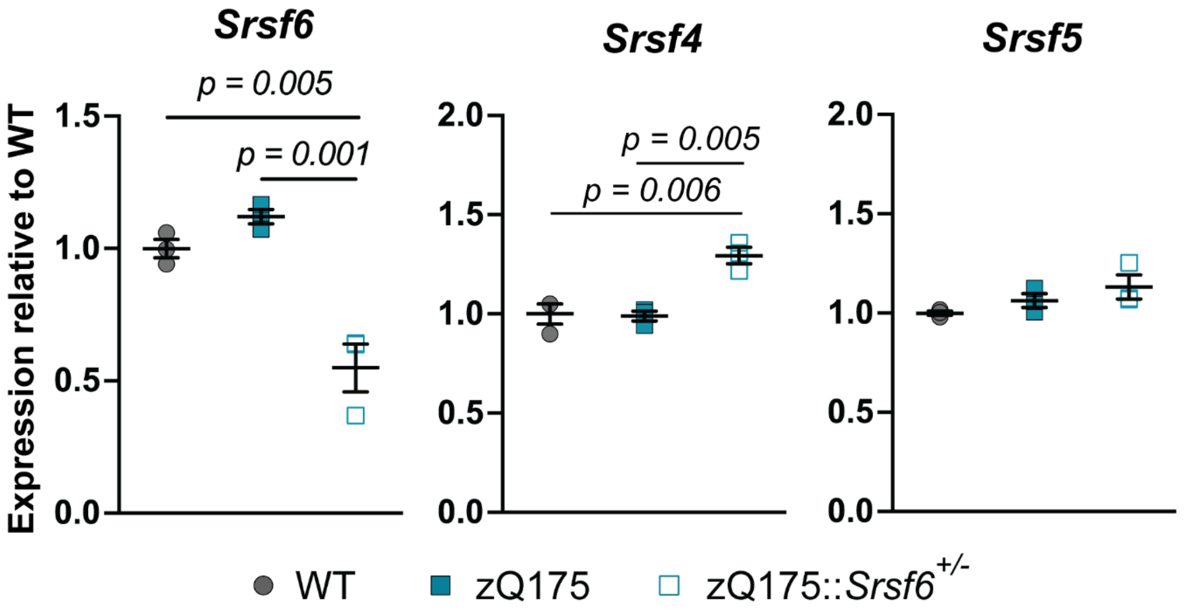

Supplementary Figure S3. SR protein transcript levels were measured in MEFs by QuantiGene. *Srsf6* levels were 50 % lower in zQ175::*Srsf6*$^{+/-}$ MEFs compared to WT or zQ175. *Srsf4* levels were increased in zQ175::*Srsf6*$^{+/-}$ MEFs compared to WT and there was no change in *Srsf5* levels. n = 3/genotype. Data were analysed by one-way ANOVA with Bonferroni correction for multiple pairwise comparisons, ***p<0.001. Test statistics can be found in Supplementary Table S13.

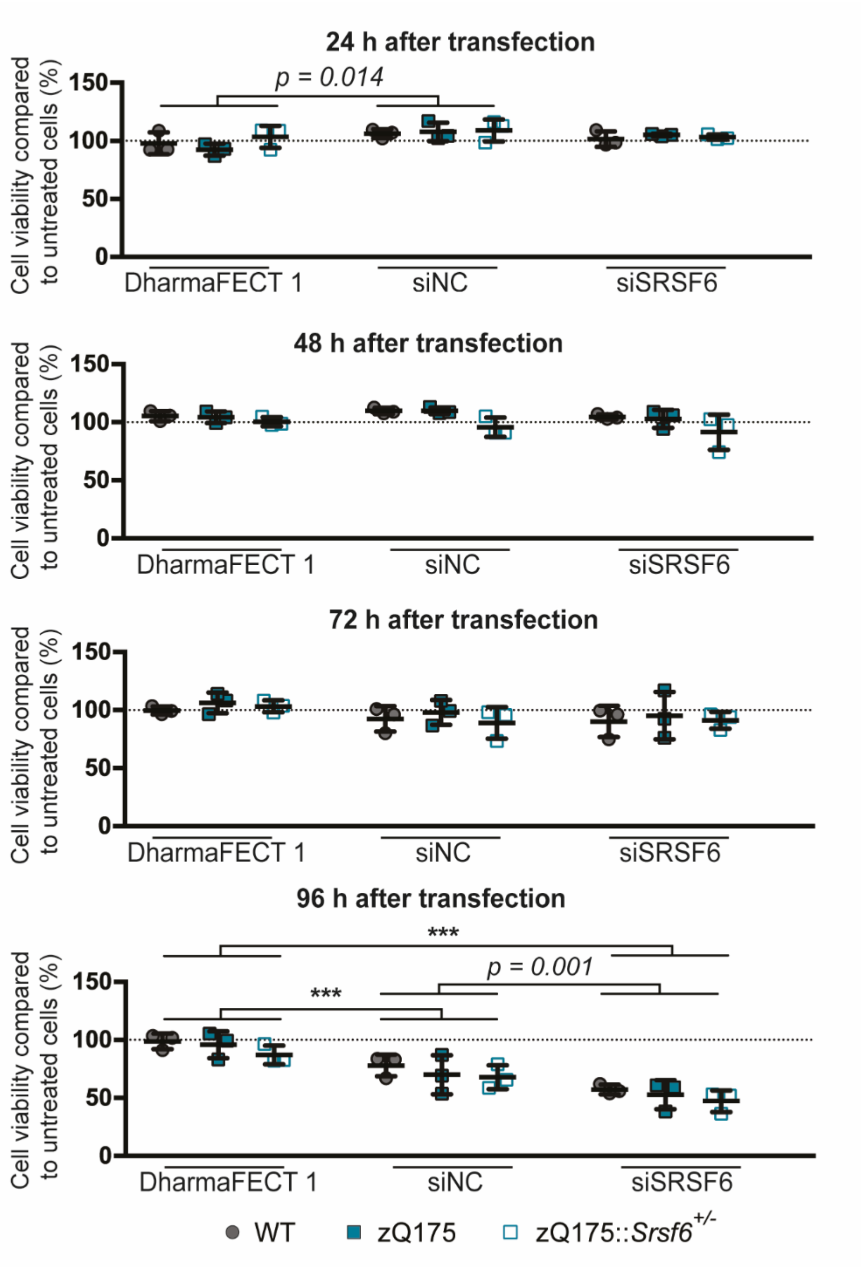

Supplementary Fig. S4. Cell viability analysis using alamarBlue in MEFs following siRNA application. siRNAs were transfected into MEFs and cell viability was assessed 24, 48, 72 and 96 hours later. In Fig. 4b, these data were combined for each genotype. n = 3 biological replicates / genotype. Statistical analysis was by two-way ANOVA, ***p > 0.001. Test statistics can be found in Supplementary Table S14. WT = wild type.

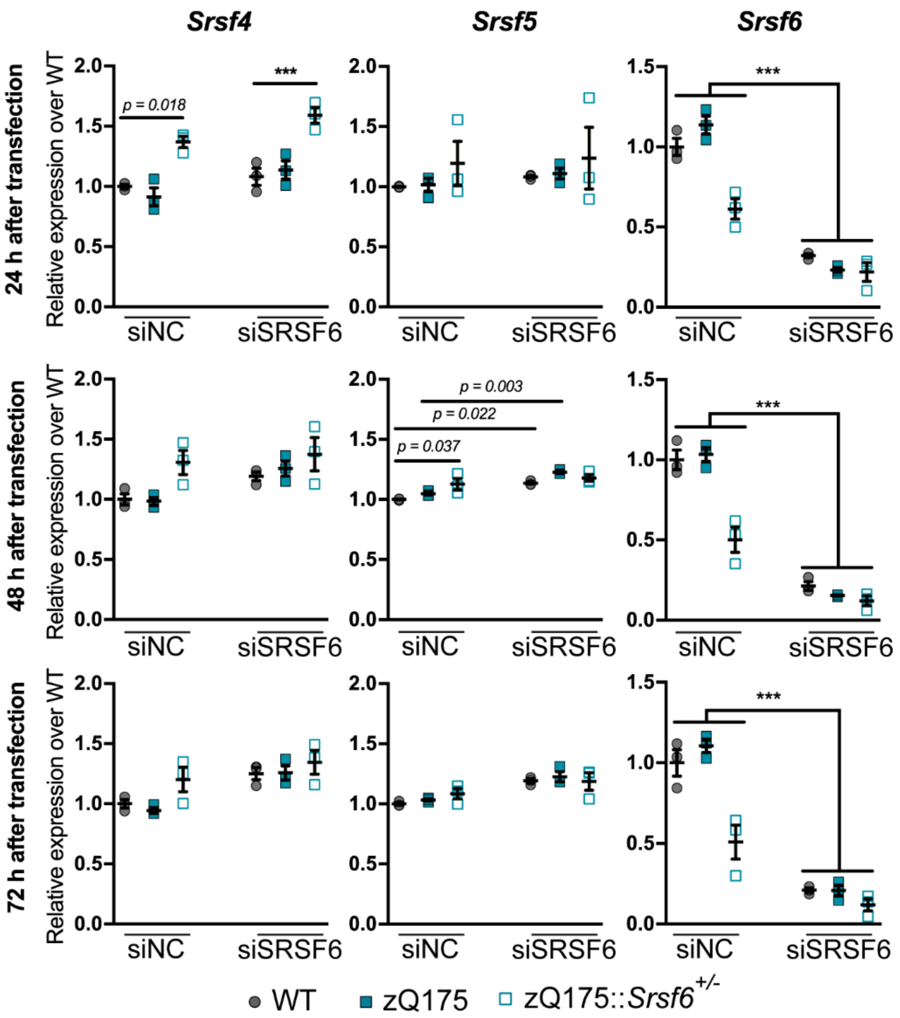

Supplementary Fig. S5. Measurement of *Srsf4*, *Srsf5 and Srsf6* transcripts in wild type, zQ175 and zQ175::*Srsf6*$^{+/-}$ MEFs after transfection with an siRNA targeting *Srsf6* (siSRSF6). *Srsf4* mRNA levels were increased in zQ175::*Srsf6*$^{+/-}$ MEFs compared to wild type at 24 h post transfection with either siNC or siSRSF6. *Srsf5* mRNAs were increased in zQ175::*Srsf6* MEFs at 48 h post-transfection, and in wild type and zQ175 MEFs 48 h after siSRSF6 transfection. n = 3/genotype. *Srsf6* mRNAs were decreased in wild type, zQ175 and zQ175::*Srsf6* MEFs at 24, 48 and 72 h post-transfection with siSRSF6 and not with siNC. n = 3/genotype. Data for *Srsf4* and *Srsf5* were analysed by one-way ANOVA with Bonferroni correction for multiple pairwise comparisons. Data for *Srsf6* were analysed by two-way ANOVA, ***$p<0.001$. Test statistics can be found in Supplementary Tables S15 and S16. WT = wild type.

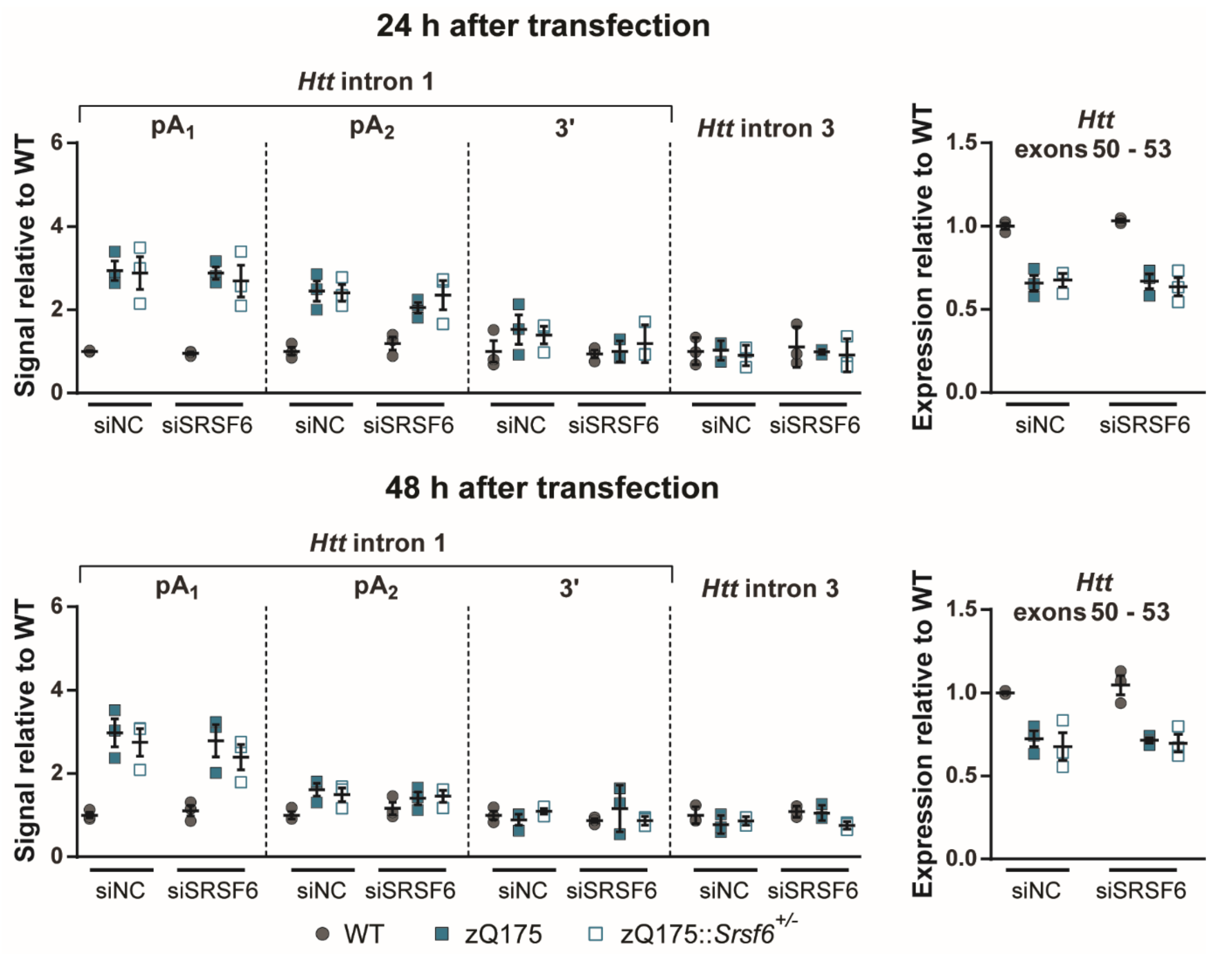

Supplementary Fig. S6. Measurement of *Httexon1* and *Htt* transcripts in WT, zQ175 and zQ175::*Srsf6*$^{+/-}$ MEFs 24 and 48 h after transfection with an siRNA targeting *Srsf6* (siSRSF6). QuantiGene analysis was used to measure the levels of *Httexon1* and *Htt* mRNA following siRNA transfection. No difference was observed in the level of either *Htt* isoform 24 or 48 h after transfection. n = 3 biological replicates / genotype. Statistical analysis was by two-way ANOVA, ***p < 0.001. Test statistics can be found in Supplementary Tables S17 and S18.

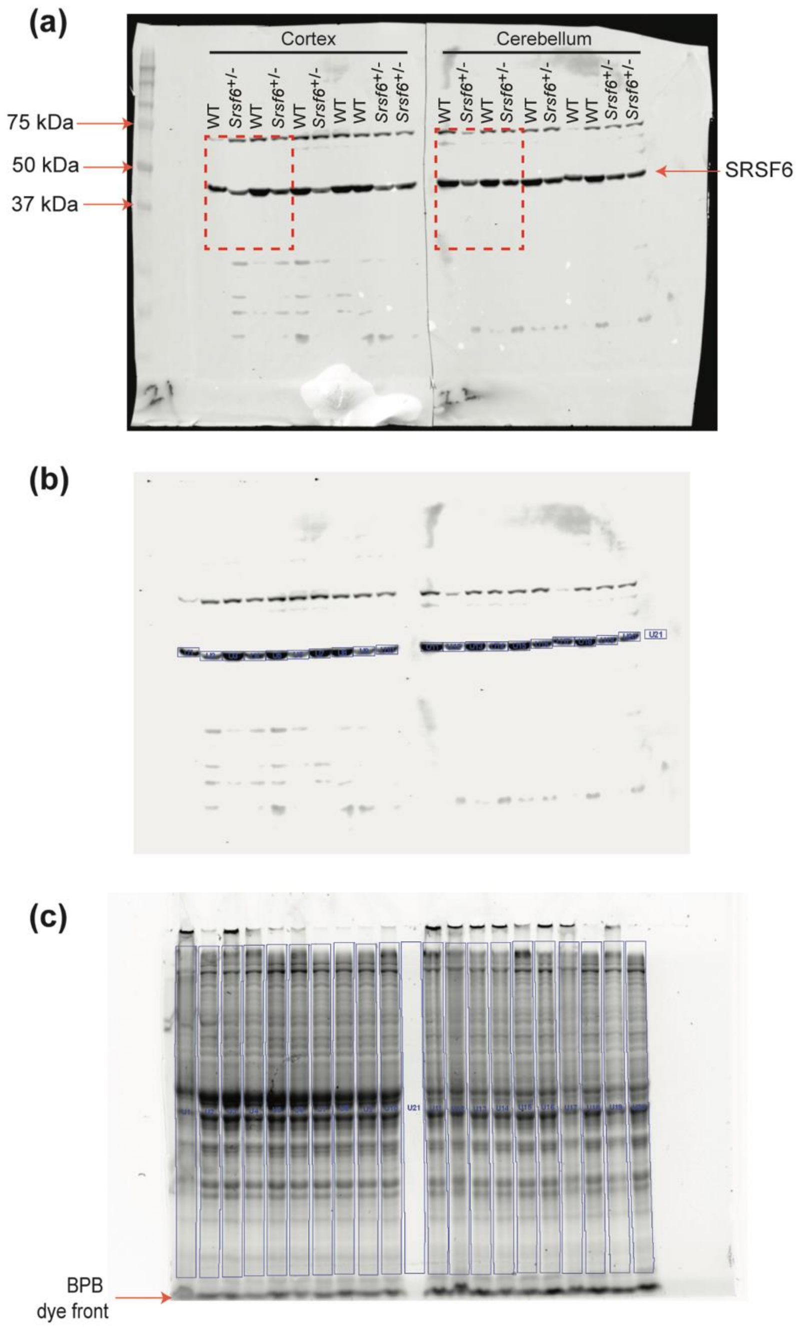

Supplementary Figure S7. SRSF6 protein was measured in 2 month old mouse cortex and cerebellum from WT and *Srsf6*$^{+/-}$ mice. (a) Colorimetric image showing an annotated protein size standard (Precision Plus Protein Dual Color Standard from BioRad) superimposed onto an exposed nitrocellulose membrane immunoblotted for SRSF6 protein from mouse brain. Genotypes (WT or *Srsf6*$^{+/-}$) and brain region (cerebellum or cortex) are annotated. The SRSF6 band resolved between the 37 kDa and 50 kDa bands of the size standard. The area within the red dashed box was cropped for Fig 1 (f-g). **(b)** SRSF6 protein was quantified using the areas in the blue boxes. **(c)** Total protein loading control was quantified using the area in the blue box. The bromophenol (BPB) dye front is annotated at the bottom.

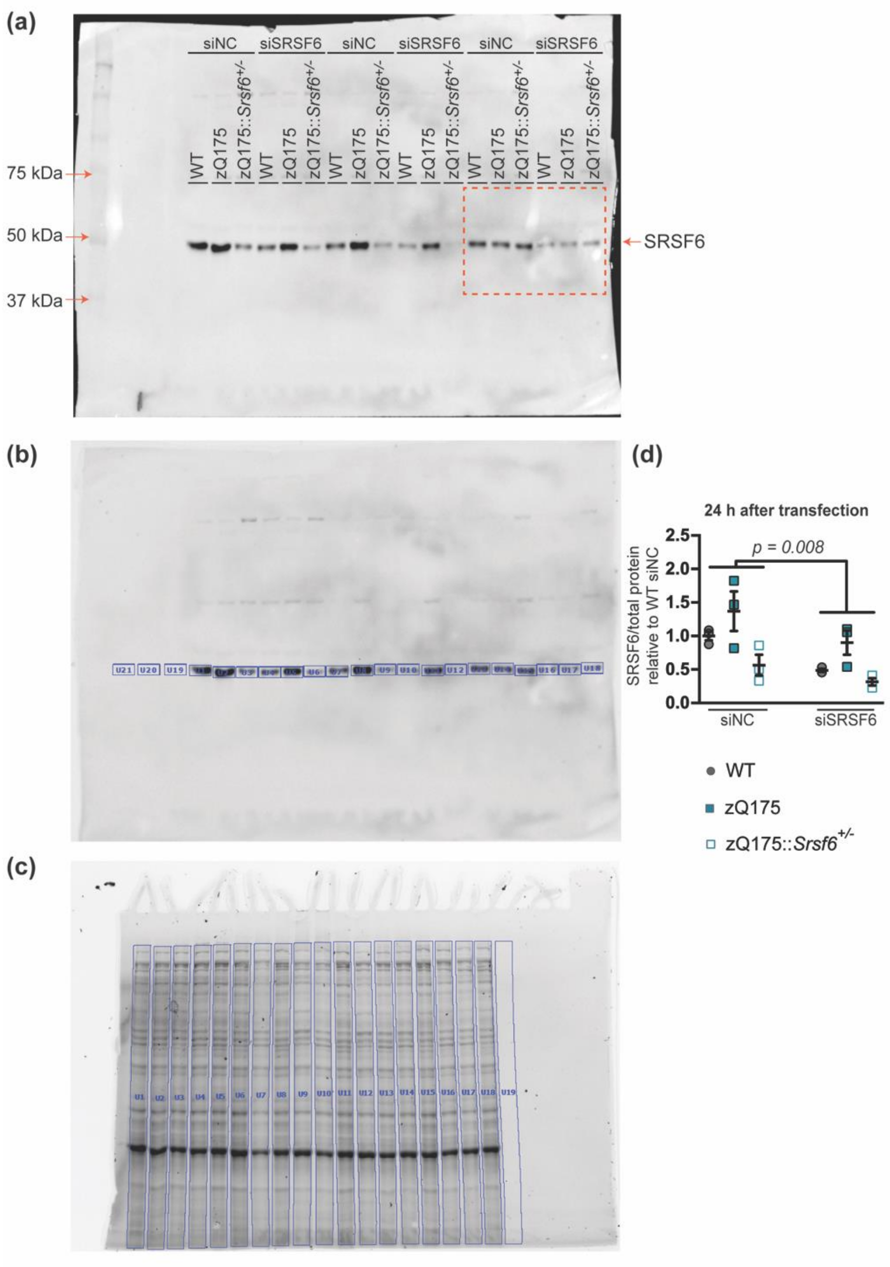

Supplementary Figure S8. (a) Colorimetric image showing an annotated protein size standard (Precision Plus Protein Dual Color Standard from BioRad) superimposed onto an exposed nitrocellulose membrane immunoblotted for SRSF6 protein from MEFs 24 h after siRNA transfection. Genotypes (WT zQ175 het or zQ175 het:: *Srsf6*$^{+/-}$) and the applied siRNA (siNC or siSRSF6) are annotated. The SRSF6 band resolved between the 37 kDa and 50 kDa bands of the size standard. The area within the red dashed box was cropped for Fig. 4d. **(b)** SRSF6 protein was quantified using the areas in the blue boxes. **(c)** Total protein loading control was quantified using the area in the blue box. **(d)** Quantification of SRSF6 protein from MEFs 24 h after siRNA transfection. n = 3 biological replicates / genotype. Statistical analysis was by two-way ANOVA. Test statistics can be found in Supplementary Table S19.

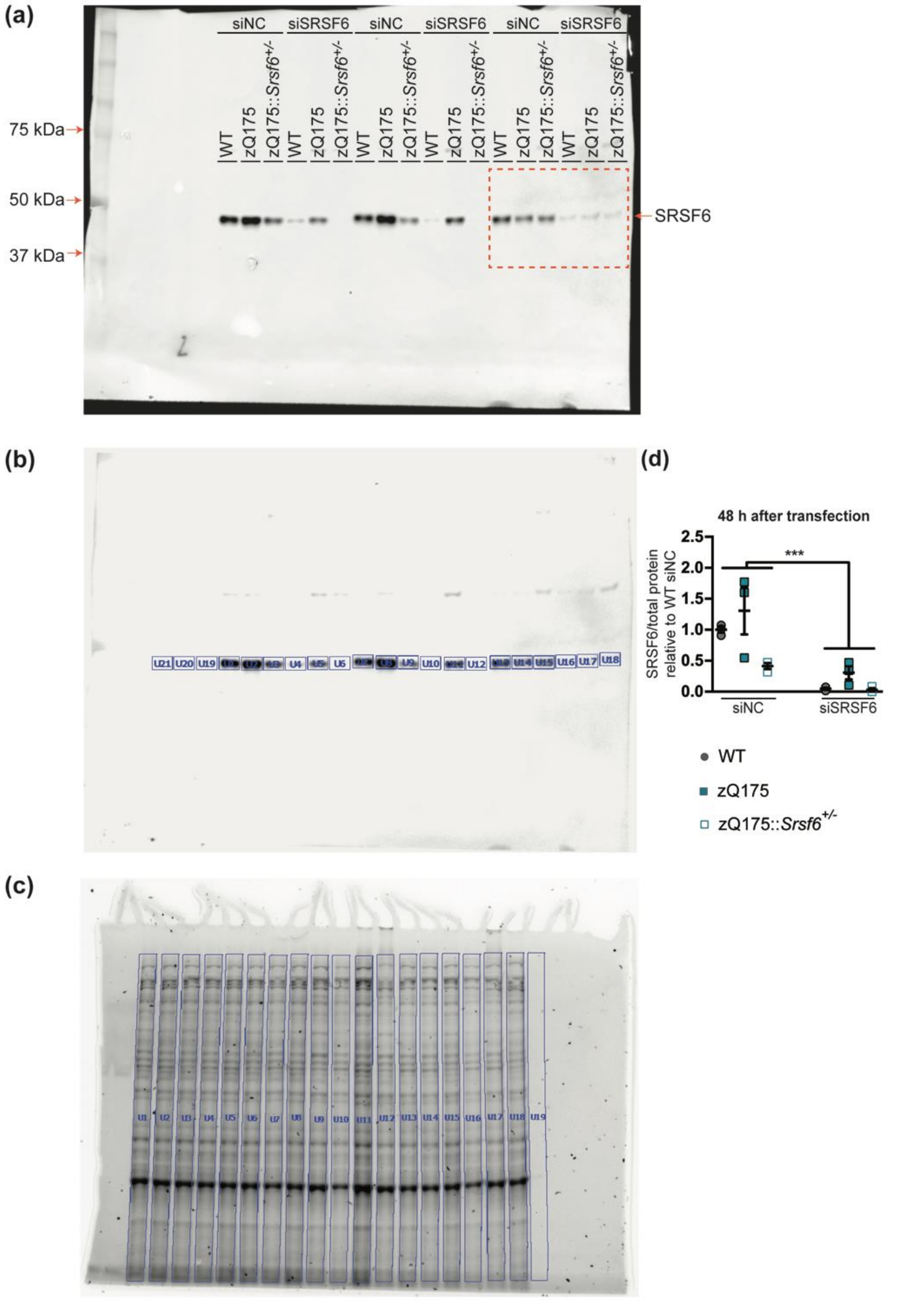

Supplementary Figure S9. (a) Colorimetric image showing an annotated protein size standard (Precision Plus Protein Dual Color Standard from BioRad) superimposed onto an exposed nitrocellulose membrane immunoblotted for SRSF6 protein from MEFs 48 h after siRNA transfection. Genotypes (WT zQ175 or zQ175::*Srsf6*$^{+/-}$) and the applied siRNA (siNC or siSRSF6) are annotated. The SRSF6 band resolved between the 37 kDa and 50 kDa bands of the size standard. The area within the red dashed box was cropped for Fig. 4d. **(b)** SRSF6 protein was quantified using the areas in the blue boxes. **(c)** Total protein loading control was quantified using the area in the blue box. **(d)** Quantification of SRSF6 protein from MEFs 48 h after siRNA transfection. n = 3 biological replicates / genotype. Statistical analysis was by two-way ANOVA. ***$p < 0.001$. Test statistics can be found in Supplementary Table S19.

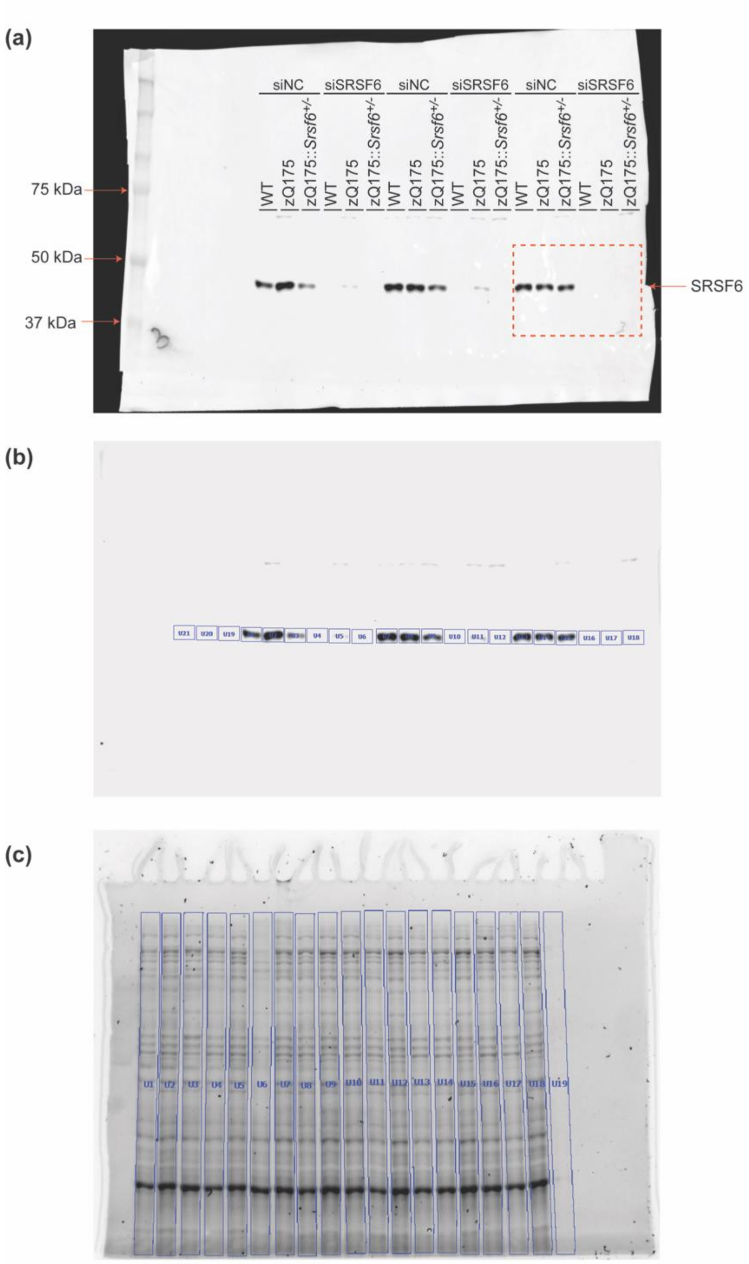

Supplementary Figure S10. (a) Colorimetric image showing an annotated protein size standard (Precision Plus Protein Dual Color Standard from BioRad) superimposed onto an exposed nitrocellulose membrane immunoblotted for SRSF6 protein from MEFs 72 h after siRNA transfection. Genotypes (WT zQ175 or zQ175::*Srsf6*$^{+/-}$) and the applied siRNA (siNC or siSRSF6) are annotated. The SRSF6 band resolved between the 37 kDa and 50 kDa bands of the size standard. The area within the red dashed box was cropped for Fig. 4d. **(b)** SRSF6 protein was quantified using the areas in the blue boxes. **(c)** Total protein loading control was quantified using the area in the blue box. Quantification graphed in Fig. 4d.

Supplementary Table S1. QuantiGene probes used for the detection of *Htt* and SR protein transcripts. House-keeping genes are in grey text.

Transcript/ Gene Symbol	Transcript/Gene name	Accession Number	Probe set region
Htt intron 1 pA$_1$	*Huntingtin – Intron 1 – poly(A)$_1$*	*GS03082**	
Htt intron 1 pA$_2$	*Huntingtin – Intron 1 – poly(A)$_2$*	*GS03084**	
Htt intron 1 3'	*Huntingtin – Intron 1 – 3'*	*GS03085**	
Htt intron 3	*Huntingtin – Intron 3*	*GS03083**	
HTT-FL (exons 50-53)	*Full length Huntingtin mRNA*	*NM010414*	*6901-7433*
Srsf6 5'	*Splicing factor, arginine/serine-rich 6 – 5'*	*NM026499*	*15-328*
Srsf6 3'	*Splicing factor, arginine/serine-rich 6 – 3'*	*NM026499*	*3054-3488*
Srsf4	*Splicing factor, arginine/serine-rich 4*	*NM020587*	*122-549*
Srsf5	*Splicing factor, arginine/serine-rich 5*	*NM009159*	*180-575*
Canx	*Calnexin*	*NM007597*	*76-727*
Rpl13a	*Ribosomal Protein L13a*	*NM009438*	*2-467*
Ubc	*Ubiquitin C*	*NM019629*	*113-676*
Atp5b	*ATP synthase subunit Beta*	*NM016774*	*22-409*
Eif4a2	*Eukaryotic translation initiation factor 4A2*	*NM013506*	*710-1271*

*QuantiGene accession numbers

Supplementary Table S2. Taqman assays used to measure expression of SR protein transcripts in mouse cortex. Housekeeping genes are in grey text.

Transcript/ Gene Symbol	Transcript/Gene name	Taqman Assay ID	Accession Number	Exon-exon junction
Srsf6 5'	*Splicing factor, arginine/serine-rich 6 – 5'*	*Mm00471474*	*NM026499*	*1-2*
Srsf6 3'	*Splicing factor, arginine/serine-rich 6 – 3'*	*Mm01239916*	*NM026499*	*4-5*
Srsf4	*Splicing factor, arginine/serine-rich 4*	*Mm00491080*	*NM020587*	*4-5*
Srsf5	*Splicing factor, arginine/serine-rich 5*	*Mm00833629*	*NM009159*	*2-3*
Canx	*Calnexin*	*Mm00500330*	*NM007597*	*4-5*
Ubc	*Ubiquitin C*	*Mm02525934*	*NM019629*	*2*
Atp5b	*ATP synthase subunit Beta*	*Mm00443967*	*NM016774*	*4-5*

Supplementary Table S3. Tissue lysate dilutions for QuantiGene plex assay.

Tissue	*Dilution of starting material (10 mg/600 μL)*	*Final input (μg/μL)*
Cortex	*1:2*	*8.25*
Striatum	*2:3*	*11*
Hippocampus	*1:2*	*8.25*
Cerebellum	*1:2*	*8.25*

Supplementary Table S4. *t*-test for Fig. 1.

	**Assay**	**Tissue**	**Statistic**
(b)	*Srsf6* exons 1 – 2 mRNA levels	Cortex	$t(9) = 3.273$, $p = 0.005$
	Srsf6 exons 4 – 5 mRNA levels	Cortex	$t(9) = 5.959$, $p < 0.001$
	Srsf4 mRNA levels	Cortex	$t(9) = 0.775$, $p = 0.458$
	Srsf5 mRNA levels	Cortex	$t(9) = -0.402$ $p = 0.697$
(c)	SRSF6 protein levels	Cerebellum	$t(8) = 4.263$, $p = 0.003$
	SRSF6 protein levels	Cortex	$t(8) = 5.083$, $p = 0.001$

Supplementary Table S5. *F*-test values for Fig. 2.

(c)	*Htt* intron 1 pA_1	Cortex	$F(3,20) = 122.762$, $p < 0.001$
	Htt intron 1 pA_2	Cortex	$F(3,20) = 86.659$, $p < 0.001$
	Htt intron 1 3’	Cortex	$F(3,20) = 7.369$, $p = 0.002$
	Htt intron 3	Cortex	$F(3,20) = 4.222$, $p = 0.018$
	Htt intron 1 pA_1	Striatum	$F(3,20) = 134.228$, $p < 0.001$
	Htt intron 1 pA_2	Striatum	$F(3,20) = 55.248$, $p < 0.001$
	Htt intron 1 3’	Striatum	$F(3,19) = 3.484$, $p = 0.036$
	Htt intron 3	Striatum	$F(3,20) = 4.672$, $p = 0.013$
	Htt intron 1 pA_1	Hippocampus	$F(3,20) = 65.822$, $p < 0.001$
	Htt intron 1 pA_2	Hippocampus	$F(3,19) = 37.59$, $p < 0.001$
	Htt intron 1 3’	Hippocampus	$F(3,19) = 4.106$, $p = 0.021$
	Htt intron 3	Hippocampus	$F(3,19) = 9.579$, $p < 0.001$
	Htt intron 1 pA_1	Cerebellum	$F(3,20) = 9.586$, $p < 0.001$
	Htt intron 1 pA_2	Cerebellum	$F(3,20) = 6.991$, $p = 0.002$
	Htt intron 1 3’	Cerebellum	$F(3,20) = 1.705$, $p = 0.198$
	Htt intron 3	Cerebellum	$F(3,20) = 5.569$, $p = 0.006$
(d)	Exons 50 - 53	Cortex	$F(3,19) = 93.59$, $p < 0.001$
	Exons 50 - 53	Striatum	$F(3,20) = 4.796$, $p = 0.011$
	Exons 50 - 53	Hippocampus	$F(3,20) = 0.062$, $p = 0.979$
	Exons 50 - 53	Cerebellum	$F(3,18) = 8.425$, $p = 0.01$

Supplementary Table S6. *F*-test values for Fig 3b.

**Assay**	**Statistic**
Htt intron 1 pA$_1$	$F(2,8) = 23.49$, $p = 0.001$
Htt intron 1 pA$_2$	$F(2,8) = 12.2$, $p = 0.008$
Htt intron 1 3’	$F(2,8) = 0.683$, $p = 0.54$
Htt intron 3	$F(2,8) = 2.512$, $p = 0.161$
Htt exons 50 - 53	$F(2,8) = 6.904$, $p = 0.028$

Supplementary Table S7. *F*-test and *t*-test values for Fig. 3c,d.

**Assay**	**Statistic**
Htt WT allele	$F(2,8) = 9.215$, $\mathrm{p} = 0.015$
Htt zQ175 knock-in allele	$t(4) = -0.179$, $p < 0.867$

Supplementary Table S8. *F*-test values for Fig 4b.

**Timepoint**	**Statistic**
24 h	$F(2,26) = 4.908$, $p = 0.016$
48 h	$F(2,26) = 1.278$, $p = 0.297$
72 h	$F(2,26) = 3.008$, $p = 0.068$
96 h	$F(2,26) = 36.972$, $p < 0.001$

Supplementary Table S9. *F*-test values for Fig. 4c.

**Timepoint**	**Factor**	**Statistic**
72 h after transfection	Treatment	$F(1,12) = 193.311$, $p < 0.001$
	Genotype	$F(2,12) = 18.385$, $p < 0.001$
	Interaction	$F(2,12) = 9.628$, $p = 0.003$

Supplementary Table S10. *F*-test values for Fig. 5 72 h after transfection.

Assay	Factor	Statistic
Htt intron 1 pA_1	Treatment	$F(1,12) = 0.024$, $p = 0.88$
	Genotype	$F(2,12) = 9.065$, $p = 0.004$
	Interaction	$F(2,12) = 0.318$, $p = 0.734$
Htt intron 1 pA_2	Treatment	$F(1,12) = 0.009$, $p = 0.926$
	Genotype	$F(2,12) = 3.114$, $p = 0.081$
	Interaction	$F(2,12) = 0.081$, $p = 0.922$
Htt intron 1 3’	Treatment	$F(1,12) = 0.108$, $p = 0.748$
	Genotype	$F(2,12) = 1.125$, $p = 0.357$
	Interaction	$F(2,12) = 0.978$, $p = 0.404$
Htt intron 3	Treatment	$F(1,12) = 0.616$, $p = 0.448$
	Genotype	$F(2,12) = 2.555$, $p = 0.119$
	Interaction	$F(2,12) = 0.425$, $p = 0.663$
Htt exons 50 - 53	Treatment	$F(1,12) = 2.991$, $p = 0.109$
	Genotype	$F(2,12) = 24.177$, $p < 0.001$
	Interaction	$F(2,12) = 0.026$, $p = 0.975$

Supplementary Table S11. *t*-test values for Supplementary Fig. S1.

**Assay**	**Statistic**
Srsf6 5’	$t(8) = 3.601$, $p = 0.007$
Srsf6 3’	$t(8) = 5.548$, $p = 0.001$
Srsf4	$t(8) = 0.773$, $p = 0.462$
Srsf5	$t(8) = 0.196$, $p = 0.85$

Supplementary Table S12. *F*-test values for Supplementary Fig. S2.

**Assay**	**Tissue**	**Statistic**
Srsf6 5’	Cortex	$F(3,20) = 120.02$, $p < 0.001$
Srsf6 5’	Striatum	$F(3,20) = 21.864$, $p < 0.001$
Srsf6 5’	Hippocampus	$F(3,19) = 81.29$, $p < 0.001$
Srsf6 5’	Cerebellum	$F(3,20) = 111.184$, $p < 0.001$
Srsf6 3’	Cortex	$F(3,20) = 128.357$, $p < 0.001$
Srsf6 3’	Striatum	$F(3,20) = 100.222$, $p < 0.001$
Srsf6 3’	Hippocampus	$F(3,18) = 96.3$, $p < 0.001$
Srsf6 3’	Cerebellum	$F(3,18) = 579.114$, $p < 0.001$
Srsf4	Cortex	$F(3,19) = 9.243$, $p < 0.001$
Srsf4	Striatum	$F(3,19) = 4.546$, $p = 0.015$
Srsf4	Hippocampus	$F(3,20) = 4.224$, $p = 0.017$
Srsf4	Cerebellum	$F(3,19) = 2.811$, $p = 0.067$
Srsf5	Cortex	$F(3,20) = 0.318$, $p = 0.812$
Srsf5	Striatum	$F(3,20) = 0.658$, $p = 0.587$
Srsf5	Hippocampus	$F(3,20) = 2.19$, $p = 0.121$
Srsf5	Cerebellum	$F(3,17) = 0.222$, $p = 0.88$

Supplementary Table S13. *F*-test values for Supplementary Fig. S3.

**Assay**	**Statistic**
Srsf6	$F(2,8) = 26.976$, $p = 0.001$
Srsf4	$F(2,8) = 18.577$, $p < 0.003$
Srsf5	$F(2,8) = 2.622$, $p = 0.152$

Supplementary Table S14. *F*-test values for Supplementary Fig. S4.

Timepoint	Factor	Statistic
24 h after transfection	Treatment	$F(2,18) = 5.992$, $p = 0.01$
	Genotype	$F(2,18) = 2.308$, $p = 0.128$
	Interaction	$F(4,18) = 1.671$, $p = 0.2$
48 h after transfection	Treatment	$F(2,18) = 1.527$, $p = 0.244$
	Genotype	$F(2,18) = 3.89$, $p = 0.039$
	Interaction	$F(4,18) = 0.724$, $p = 0.2$
72 h after transfection	Treatment	$F(2,18) = 2.471$, $p = 0.113$
	Genotype	$F(2,18) = 0.691$, $p = 0.514$
	Interaction	$F(4,18) = 0.086$, $p = 0.986$
96 h after transfection	Treatment	$F(2,18) = 35.493$, $p < 0.001$
	Genotype	$F(2,18) = 2.348$, $p = 0.124$
	Interaction	$F(4,18) = 0.086$, $p = 0.986$

Supplementary Table S15. *F*-test values for *Srsf4* and *Srsf5* from Supplementary Fig. S5.

24 h after transfection	*Srsf4*	$F(5,12) = 16.512$, $p < 0.001$
	Srsf5	$F(5,12) = 0.514$, $p = 0.761$
48 h after transfection	*Srsf4*	$F(5,12) = 4.162$, $p = 0.02$
	Srsf5	$F(5,12) = 12.357$, $p < 0.001$
72 h after transfection	*Srsf4*	$F(5,12) = 5.355$ $p = 0.008$
	Srsf5	$F(5,12) = 5.447$, $p = 0.008$

Supplementary Table S16. *F*-test values for *Srsf6* from Supplementary Fig. S5.

Assay	Time point	Factor	Statistic
Srsf6	24 h after transfection	Treatment	$F(1,12) = 287.766$, $p < 0.001$
		Genotype	$F(2,12) = 19.535$, $p < 0.001$
		Interaction	$F(2,12) = 14.544$, $p = 0.001$
	48 h after transfection	Treatment	$F(1,12) = 25.046$, $p < 0.001$
		Genotype	$F(2,12) = 681.719$, $p < 0.001$
		Interaction	$F(2,12) = 15.703$, $p < 0.001$

Supplementary Table S17. *F*-test values for Supplementary Fig. S6 24 h after transfection.

Assay	Factor	Statistic
Htt intron 1 pA_1	Treatment	$F(1,12) = 0.235$, $p = 0.636$
	Genotype	$F(2,12) = 37.275$, $p < 0.001$
	Interaction	$F(2,12) = 0.05$, $p = 0.951$
Htt intron 1 pA_2	Treatment	$F(1,12) = 0.226$, $p = 0.615$
	Genotype	$F(2,12) = 22.244$, $p < 0.001$
	Interaction	$F(2,12) = 0.958$, $p = 0.411$
Htt intron 1 3’	Treatment	$F(1,12) = 1.878$, $p = 0.196$
	Genotype	$F(2,12) = 1.142$, $p = 0.351$
	Interaction	$F(2,12) = 0.517$, $p = 0.609$
Htt intron 3	Treatment	$F(1,12) = 0.032$, $p = 0.86$
	Genotype	$F(2,12) = 0.333$, $p = 0.723$
	Interaction	$F(2,12) = 0.074$, $p = 0.929$
Htt exons 50 - 53	Treatment	$F(1,12) = 0.001$, $p = 0.97$
	Genotype	$F(2,12) = 54.692$, $p < 0.001$
	Interaction	$F(2,12) = 0.435$, $p = 0.657$

Supplementary Table S18. *F*-test values for Supplementary Fig. S6 48 h after transfection.

Assay	Factor	Statistic
Htt intron 1 pA_1	Treatment	$F(1,12) = 0.384$, $p = 0.547$
	Genotype	$F(2,12) = 23.668$, $p < 0.001$
	Interaction	$F(2,12) = 0.34$, $p = 0.718$
Htt intron 1 pA_2	Treatment	$F(1,12) = 0.052$, $p = 0.823$
	Genotype	$F(2,12) = 5.328$, $p = 0.022$
	Interaction	$F(2,12) = 0.816$, $p = 0.465$
Htt intron 1 3'	Treatment	$F(1,12) = 0.052$, $p = 0.823$
	Genotype	$F(2,12) = 0.164$, $p = 0.851$
	Interaction	$F(2,12) = 1.459$, $p = 0.271$
Htt intron 3	Treatment	$F(1,12) = 1.2$, $p = 0.295$
	Genotype	$F(2,12) = 3.091$, $p = 0.083$
	Interaction	$F(2,12) = 2.04$, $p = 0.173$
Htt exons 50 - 53	Treatment	$F(1,12) = 0.212$, $p = 0.654$
	Genotype	$F(2,12) = 26.474$, $p < 0.001$
	Interaction	$F(2,12) = 0.148$, $p = 0.864$

Supplementary Table S19. *F*-test values for Supplementary Fig. S8 and S9.

	Factor	Statistic
24 h after transfection	Treatment	$F(1,12) = 10.007$, $p = 0.008$
	Genotype	$F(2,12) = 9.618$, $p = 0.003$
	Interaction	$F(2,12) = 0.4$, $p = 0.679$
48 h after transfection	Treatment	$F(1,12) = 33.478$, $p < 0.001$
	Genotype	$F(2,12) = 6.265$, $p = 0.014$
	Interaction	$F(2,12) = 2.129$, $p = 0.162$
